# MCL-1 inhibitors, fast-lane development of a new class of anti-cancer agents

**DOI:** 10.1186/s13045-020-01007-9

**Published:** 2020-12-11

**Authors:** Arnold Bolomsky, Meike Vogler, Murat Cem Köse, Caroline A. Heckman, Grégory Ehx, Heinz Ludwig, Jo Caers

**Affiliations:** 1 Wilhelminen Cancer Research Institute, Wilhelminenspital, Vienna, Austria; 2 Department of Clinical Hematology, GIGA-I3, University of Liège, CHU De Liège, 35, Dom Univ Sart Tilman B, 4000 Liège, Belgium; 3 Institute for Experimental Cancer Research in Pediatrics, Goethe-University, Frankfurt, Germany; 4 Institute for Molecular Medicine Finland—FIMM, HiLIFE—Helsinki Institute of Life Science, iCAN Digital Cancer Medicine Flagship, University of Helsinki, Helsinki, Finland

**Keywords:** Myeloid cell leukemia 1, MCL-1, BCL-2, Dependency, Inhibitor, Apoptosis, Cancer, Leukemia, Myeloma, Lymphoma, Melanoma

## Abstract

Cell death escape is one of the most prominent features of tumor cells and closely linked to the dysregulation of members of the Bcl-2 family of proteins. Among those, the anti-apoptotic family member myeloid cell leukemia-1 (MCL-1) acts as a master regulator of apoptosis in various human malignancies. Irrespective of its unfavorable structure profile, independent research efforts recently led to the generation of highly potent MCL-1 inhibitors that are currently evaluated in clinical trials. This offers new perspectives to target a so far undruggable cancer cell dependency. However, a detailed understanding about the tumor and tissue type specific implications of MCL-1 are a prerequisite for the optimal (i.e., precision medicine guided) use of this novel drug class. In this review, we summarize the major functions of MCL-1 with a special focus on cancer, provide insights into its different roles in solid vs. hematological tumors and give an update about the (pre)clinical development program of state-of-the-art MCL-1 targeting compounds. We aim to raise the awareness about the heterogeneous role of MCL-1 as drug target between, but also within tumor entities and to highlight the importance of rationale treatment decisions on a case by case basis.

## Introduction

The intrinsic apoptosis pathway represents the most prominent cell death signaling cascade and is primarily controlled by the BCL-2 family of proteins. This can be split into pro-survival/anti-apoptotic (BCL-2, BCL-xL, MCL-1, BCL-W, BFL1), effector (BAK, BAX, BOK), BH3-only activator (BIM, BID, PUMA) and sensitizer (NOXA, BAD, BMF, BIK, Hrk) proteins [1]. The decision between cellular survival and death depends on the precise balance of these anti- and pro-apoptotic proteins. Under homeostasis, anti-apoptotic proteins bind to and sequester both BH3-only activator as well as effector molecules thereby ensuring cellular survival. In the presence of a cell death stimuli (e.g., radiation, chemotherapy), however, transcriptional upregulation as well as prost-translational modifications of BH3-only activator and/or sensitizer proteins initiate the intrinsic apoptosis pathway. BH3-only activator and sensitizer proteins directly bind to anti-apoptotic BCL-2 family members with high-affinity which mediates the release of sequestered effector molecules (BAK and BAX). Moreover, free BH3-only activator proteins directly bind to and activate BAK/BAX, which leads to BAK/BAX homo-oligomerization, mitochondrial outer membrane permeabilization (MOMP), release of cytochrome c into the cytosol and finally caspase activation [1].

In healthy cells, the balance between death and pro-survival signals is a tightly regulated process that depends on tissue location and age [1–3]. In adults, non-hematopoietic organs (e.g., brain, heart and kidneys) are characterized by a low expression of essential effector molecules of apoptosis (BAK and BAX) and are thus apoptosis refractory [2]. On the contrary, hematopoietic organs are typically “primed” for apoptosis, i.e., there is only a minimal excess of anti- over pro-apoptotic proteins that enables cells of the hematopoietic lineage to escape cell death [2]. External triggers that lead to cellular stress or damage consequently lead to the rapid induction of apoptosis in primed cells.

**Fig. 1:**
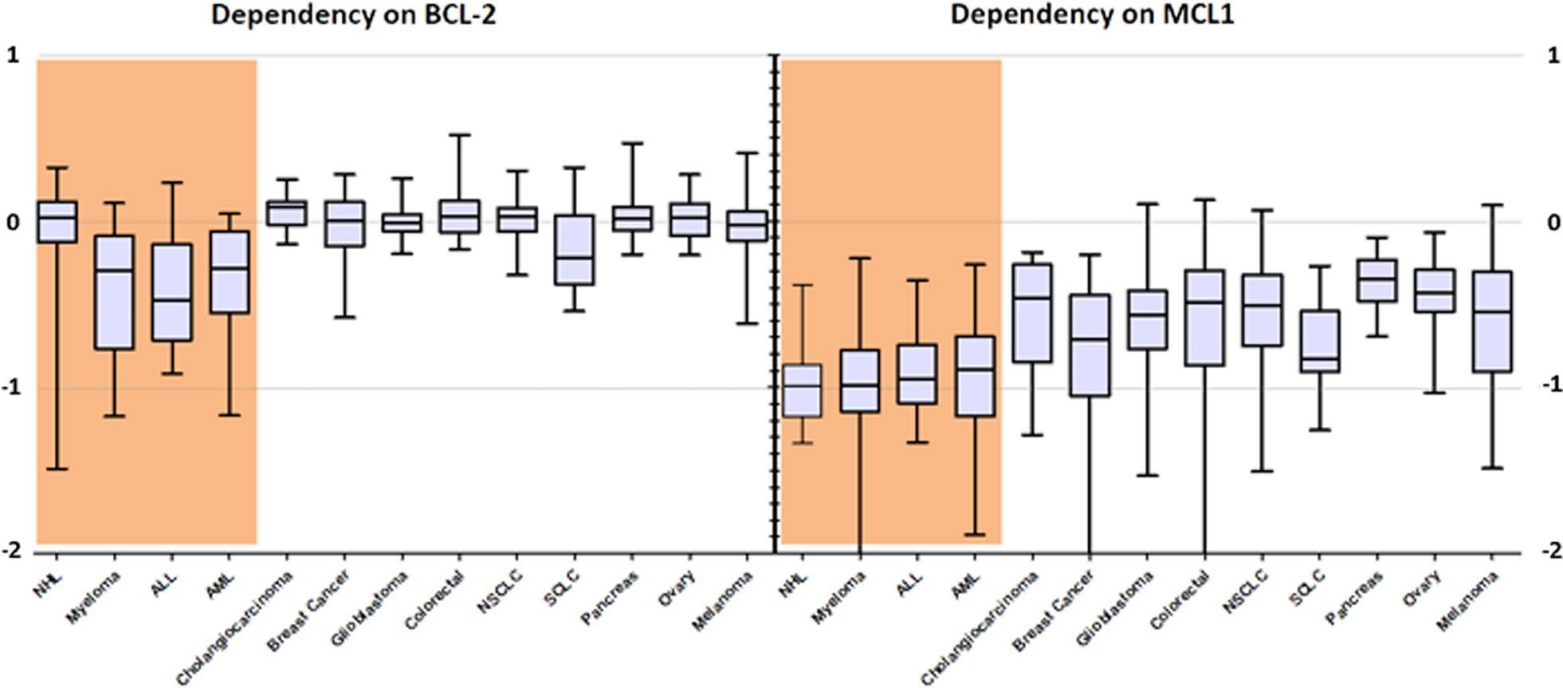
Dependencies of tumor cell lines on MCL-1. Depmap analysis indicated that knockout of MCL-1 using CRISPR/Cas9 could significantly suppress the growth of both solid and blood cancers, the latter being more affected. Lower values indicate that a gene is more likely to be dependent in a given cell line. A score of 0 indicates non-essential genes whereas a score of -1 corresponds to the median of all common essential genes. Suppression of BCL-2 affected the survival of hematological tumor cell lines to a lesser extent, but had no effect on solid tumor cells (www.depmap.org)

**Fig. 2:**
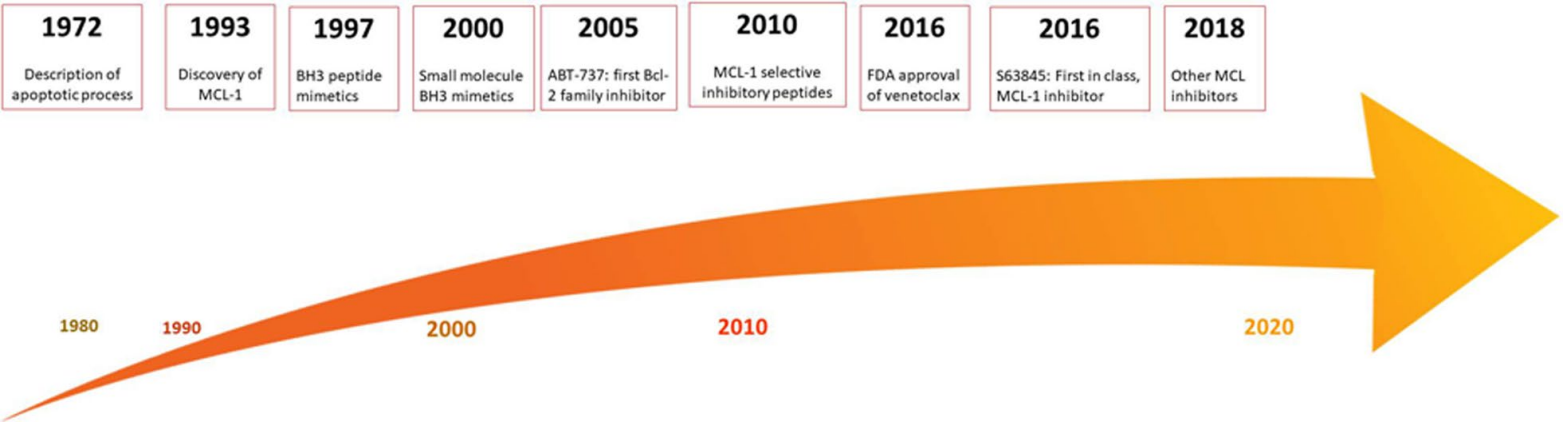
Milestones in the development of MCL-1 inhibitors

Primed cells are marked by higher proliferation rates and present some of the features of tumor cells [1]. The abilities of malignant cells to evade cell death likewise depend on individual or multiple pro-survival proteins (MCL-1, BCL-2, BCL-xL) that bind to and sequester pro-apoptotic BH3-only activator as well as effector molecules [4]. The discrete pattern of BCL-2 family dependencies is, however, remarkably heterogeneous between and within distinct neoplasias. Modern approaches such as BH3 profiling and large-scale CRISPR screens were able to shed light on the prominent role of MCL-1 in tumor cell death evasion [5–7]. Figure 1 illustrates the dependencies of several cancer cell lines to MCL-1 or BCL-2 silencing using CRISPR/Cas9 (data available at depmap.org) [8]. While solid tumors only displayed dependencies toward MCL-1, hematological malignancies were dependent on both MCL-1 and BCL-2. This difference can be partially explained by an increased level of priming that is seen in hematological cells. This dependency on MCL-1 is further driven by both genetic and functional alterations in tumor cells that will be addressed in the current review and presented alongside upcoming treatment strategies that specifically target MCL-1 dependency in tumor cells. After a long journey of intensive drug development efforts (Fig. 2), potent and selective MCL-1 inhibitors were rapidly developed and are currently entering in clinical trials.

## Multi-functional roles of MCL-1

Myeloid-cell leukemia 1 (MCL-1) was discovered, isolated (and named) from the human myeloid leukemia cell line ML-1 [9]. MCL-1 was found to have close sequence similarities with BCL-2 and both genes shared “surprising” oncogenic properties: they sustained cell survival but did not promote cell proliferation [9]. Almost thirty years later BCL-2 and MCL-1 are the most prominent members of the BCL-2 family and well known for their anti-apoptotic role in health and disease.

MCL-1 shares the presence of BCL-2 homology regions (BH1-4) and a carboxy-terminal transmembrane localization domain with other pro-survival family members (e.g., BCL-2 and BCL-xL) [10]. The alpha helix of the BH3 domain is essential for direct interactions between BCL-2 family members [11]. Anti-apoptotic family members including MCL-1 form a hydrophobic groove (composed of BH1-BH3 domains), where four hydrophobic binding pockets (P1–P4) guide the interaction with hydrophobic residues (h1-h4) of BH3 domains. In addition, a conserved arginine within the BH1 domain is a key binding anchor for a conserved aspartate of BH3-only proteins [10–12].

Irrespective of these similarities between distinct pro-survival BCL-2 family members, MCL-1 differs in some key characteristics. MCL-1 is larger than other pro-survival BCL-2 family members. This is due to the presence of a N-terminal sequence rich in proline (P), glutamic acid (E), serine (S) and threonine (T) residues (PEST). The PEST sequence regulates MCL-1 turnover and the post-translational modifications (recently reviewed by Senichkin et al. [13]).

Moreover, sequence differences lead to a unique residue profile within the BH3 binding groove and a more electropositive binding surface as compared to BCL-2 and BCL-xL [12, 14]. This causes the unique binding affinity profile of MCL-1, with strong affinities for BAK, BIM, NOXA and PUMA, but only weak affinity for BMF and BAD. Direct sequestration of BAK and BIM is a key gatekeeper function of MCL-1 in the prevention of apoptosis. In turn, the BH3-only sensitizer protein NOXA specifically hinders MCL-1 from the exertion of its anti-apoptotic functions, frees pro-apoptotic molecules and thereby initiates cell death [15, 16].

The importance of MCL-1 and its anti-apoptotic functions for normal cellular development is demonstrated by numerous knockout studies. The inducible deletion of Mcl-1 during early lymphocyte differentiation increased apoptosis and arrested the development at pro-B-cell and double-negative T-cell stages. Induced deletion of Mcl-1 in mature B- and T-cell populations resulted in their rapid loss [17]. Other conditional gene knockout studies demonstrated an essential role for MCL-1 in hematopoietic stem cells, hepatocytes, neutrophils, cardiomyocytes and during neuronal development [18]. Moreover, systemic deletion of MCL-1 in murine ES cells resulted in peri-implantation embryonic lethality [19]. Strikingly, MCL-1-/-blastocysts showed a maturation delay but no increase in apoptosis [19]. A similar observation was made in MCL-1 knockout studies in cardiomyocytes, where the simultaneous inhibition of apoptosis was not able to reverse the impact on mitochondrial structure and respiration [20]. This suggests that MCL-1 plays a role in cellular health and development that goes beyond its established pro-survival functions.

Several studies support central non-apoptotic roles of MCL-1. In addition to its outer mitochondrial membrane (OMM) localization, MCL-1 was shown to reside at the inner mitochondrial membrane (IMM) where it stabilizes mitochondrial structures, mitochondrial fusion, ATP production, mitochondrial membrane potential and oxygen consumption rate [21]. Mechanistically, MCL-1 was shown to directly interact with the mitochondrial fission and fusion regulators DRP-1 and OPA1 in human pluripotent stem cells (hPSCs), thereby influencing mitochondrial dynamics [22]. These results were recently confirmed in hPSC-derived cardiomyocytes, underlining the importance of MCL-1 for mitochondrial network organization. Interestingly, treatment of cardiomyocytes with the MCL-1 specific inhibitor S63845, but not venetoclax, significantly disrupted mitochondrial homeostasis [23]. The available information therefore strongly supports the idea that MCL-1 is not only a central antagonist of cell death, but also an essential molecule for normal mitochondrial function. Additional non-apoptotic functions of MCL-1 such as a role in the DNA damage response pathway or autophagy are currently emerging [24, 25]. Although these results provide exciting novel insights into the pleiotropic functions of MCL-1, they also raise concerns about the therapeutic window for MCL-1 inhibitors. The close monitoring of patients will be key to guarantee the safety of this novel drug class, particularly in those receiving long-term treatment.

## Overview of the role of MCL-1 in cancer

Cell death evasion represents a cancer hallmark and it is therefore not surprising that several BCL-2 family members play a central role in tumor formation and survival [26]. In contrast to other Bcl-2 family members, MCL-1 is usually not involved in translocations; also mutations of MCL-1 are very infrequent (detected in approximately 1% of all cancers) [27] although they were shown to prolong MCL-1 stability and affect the activity of MCL-1 inhibitors [28]. However, MCL-1 stands out as it is one of the most frequently and highly amplified genes in human cancers [29]. The functional relevance of this frequent upregulation of MCL-1 expression was verified in multiple tumor models: importantly, amplification of the MCL-1 gene locus (1q21.2) correlates with MCL-1 dependency as evidenced by the impact of MCL-1 silencing [29]. Mice overexpressing MCL-1 in hematopoietic stem/progenitor cells are prone to malignant transformation, particularly (MYC-driven) lymphomas of the B-cell lineage [30, 31]. Accordingly, heterozygous loss of MCL-1 was sufficient to inhibit MYC-driven lymphomagenesis in > 80% of Eμ-Myc;MCL-1 + / − mice. This was linked to the anti-apoptotic role of MCL-1 as concurrent loss of the BH3-only protein(s) BIM (and to a lesser extent PUMA) reversed this phenotype [32]. Additional tumor formation studies in mice confirmed an essential role for MCL-1 in the development of acute myeloid leukemia (AML), T-cell lymphomas and breast cancer [33, 34].

The striking impact of perturbing MCL-1 dependency on tumor development underscores the need of tumor cells to sustain MCL-1 expression and stability. Due to the short half-life of MCL-1 (typically referred to as < 1 h) [36], MCL-1-dependent leukemic cells are more sensitive to chemotherapy as compared to BCL-2 dependent cells [37, 38]. Given the lack of recurrent MCL-1 mutations, tumor cells require alternative mechanisms to overcome this vulnerability in order to sustain MCL-1 activity. Multiple adaptation processes have been described that culminate in enhanced MCL-1 stability, including the deregulation (either over- or underexpression), post-translational modifications as well as mutational events of molecules involved in MCL-1 degradation and/or stabilization.

**Fig. 3:**
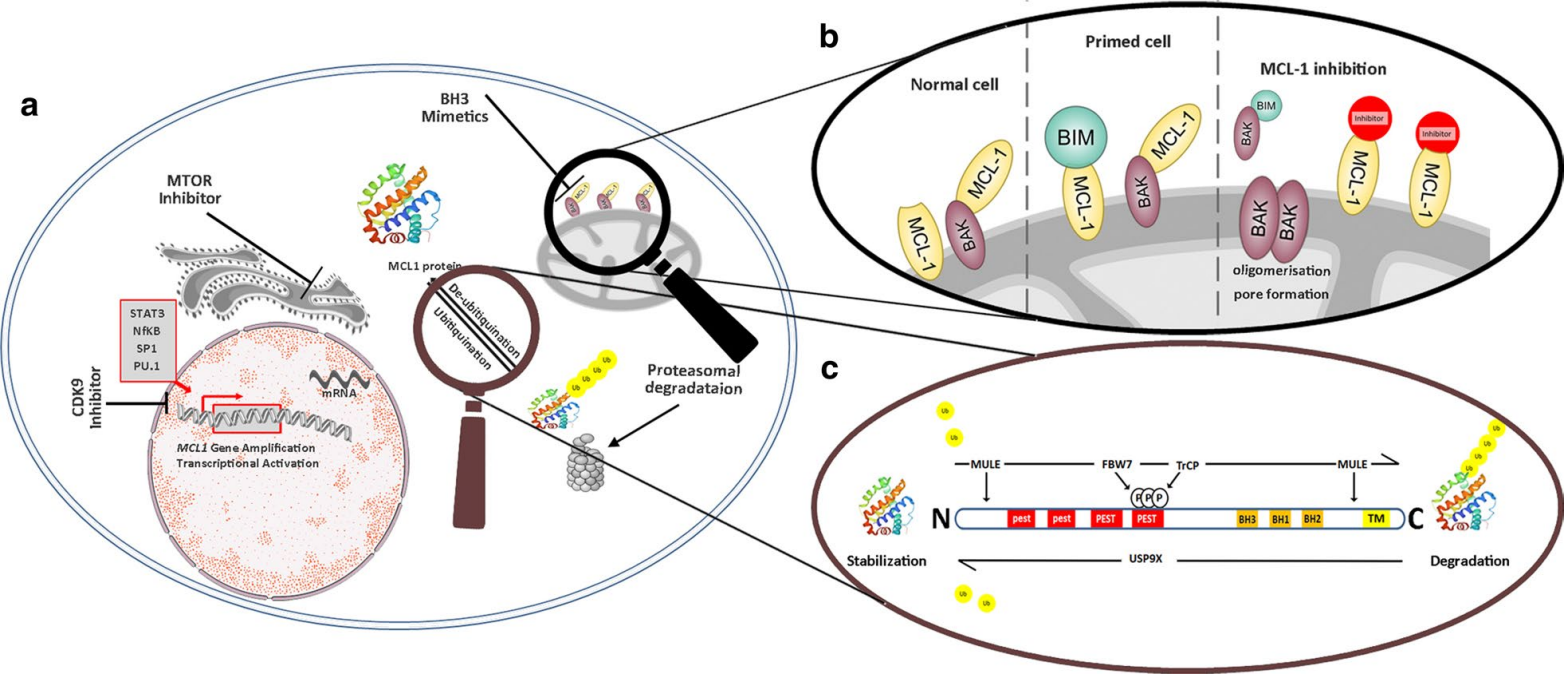
Direct and indirect targeting of MCL-1. (**a**) There are two principal approaches in the targeting of MCL-1: indirect inhibition through inhibition of transcription or translation and downregulation of MCL-1 via targeting of proteasomal degradation and direct inhibition through interruption of protein–protein interactions via small molecule inhibitors (BH3-mimetics). (**b**) At the mitochondrial membrane, MCL-1 binds the proapoptotic multidomain effector BAK to prevent cell death. In primed cells, there is only a minimal excess of anti- over pro-apoptotic proteins. A variety of cell stressors increase the expression of the proapoptotic sensors, including the BH3-only proteins (i.e., BIM, BID, PUMA, NOXA and BAD). The treatment with an MCL-1 inhibitor will liberate BAK from binding to MCL-1. BAK will oligomerize and form pores in the mitochondrial membrane leading to cytochrome c release into the cytosol and activation of the caspase cascade. (**c**) MCL-1 can be phosphorylated by several protein kinases which enables the recognition of MCL-1 by its E3 ubiquitin-ligases TrCP or FBW7. In addition, the E3 ubiquitin-ligase Mule can interact either with the C- or N-terminus of MCL-1 in a phosphorylation-independent manner. This binding can be inhibited by BIM and PUMA or increased by NOXA. Ubiquitination of MCL-1 targets it for proteasomal degradation. It can be opposed by the deubiquitinases such as USP9X that directly removes polyubiquitin chains, which results in MCL‑1 stabilization

The rapid degradation of MCL-1 in the presence of therapeutic pressure is catalyzed via the ubiquitin–proteasome pathway. MCL-1 ubiquitin ligase E3 (Mule) and the Skp1-Cullin-F box (SCF) E3 ligase substrate receptors F-box and WD repeat domain-containing 7 (FBW7) and β-TrCP are among the best studied mediators of MCL-1 ubiquitination (reviewed in Ref [13]). The binding of FBW7 to MCL-1 is facilitated via the phosphorylation of the PEST sequence of MCL-1, leading to ubiquitination and proteasomal degradation (Fig. 3). The introduction of mutations within the PEST sequence blocks this phosphorylation process and results in an increased MCL-1 half-life and increased sequestration of PUMA. Mitotic arrest induced by microtubule-targeting agents leads to the rapid phosphorylation within the PEST sequence and subsequent recognition and ubiquitination by SCF^Fbw^ [7]. However, *FBW7-deficient* tumor cells *(by deletion or loss-of-function mutations)* acquire a resistance to anti-tubulin chemotherapeutics. Moreover, these tumors are prone to therapy-induced polyploidy after mitotic slippage [39]. In T-cell acute lymphoblastic leukemia (T-ALL), *FBW7* mutations are found in up to 25% of T-ALL patients [40]. Here, SCF^Fbw^ [7] targets MCL-1 for destruction in a GSK3β dependent manner and is involved in drug resistance and tumor formation [41].

Apart from the genetic inactivation of E3 ligase components, tumor cells use dynamic processes that interfere with the proteasomal degradation of MCL-1. The prolyl isomerase Pin1 was found to directly bind to FBW7 in a phosphorylation dependent manner inducing FBW7 auto-ubiquitination and degradation. Genetic loss of Pin1 therefore leads to elevated FBW7 protein levels, loss of MCL-1 and sensitization to taxol [42]. In addition, the deubiquitinase USP9X was found to bind and remove polyubiquitin chains from MCL-1 targeted for degradation [36]. USP9X expression correlated with MCL-1 overexpression and/or poor outcome in follicular lymphoma, diffuse large B-cell lymphoma (DLBCL) and multiple myeloma (MM) [36]. Conversely, knockdown or therapeutic targeting of USP9X destabilizes MCL-1 and sensitizes tumor cells to BCL-2/BCL-xL inhibitors [36, 43]. More recently, USP24 and USP13 were likewise shown to interact, deubiquitinase and stabilize MCL-1, thereby mediating BH3 mimetic and USP9X inhibitor resistance [43, 44].

Finally, MCL-1 is not only important for tumor development and initial drug sensitivity. MCL-1 is a central driver of drug resistance against a plethora of established cancer treatments. As one might expect, MCL-1 drives resistance to BCL-2 and BCL-xL targeting compounds. Overexpression of MCL-1 promoted drug resistance of both solid tumors and hematological malignancies to various chemotherapeutic agents. Interestingly, MCL-1 in cooperation with MYC induced drug resistance in triple-negative breast cancer via upregulation of mitochondrial oxidative phosphorylation and expansion of cancer stem cells. [45] A role for metabolic pathways in MCL-1-associated drug resistance is supported by additional studies. In ALL, the mTOR inhibitor rapamycin was revealed to modulate glucocorticoid sensitivity via downregulation of MCL-1 [46]. Moreover, combined inhibition of glycolysis and OXPHOS indirectly affects MCL-1 expression and thus induces cell death in tumor cells [47].

These results illustrate the numerous implications of MCL-1 in promoting cancer survival and drug resistance. Nevertheless, our current knowledge about the regulation of BCL-2 family members in cancer clearly demonstrates that the efficacy of MCL-1 targeting strategies will depend on 1) the level of apoptotic priming and 2) the MCL-1 dependency status [6, 48]. In addition, limited single-agent activity of MCL-1 inhibitors can be boosted with rationale combination approaches such as MEK inhibitors in KRAS-mutant non-small-cell lung cancer (NSCLC) [49]. A detailed knowledge of tissue-dependent BCL-2 family dependencies in concert with treatment decisions on a case by case basis therefore offers the chance for the implementation of true precision medicine approaches.

## Solid tumors

Preclinical studies with “modern” MCL-1 inhibitors strongly pointed to hematological malignancies as prime indications for early clinical testing. However, a limited number of studies also highlighted an MCL-1 dependency in solid tumors such as NSCLC and breast cancer cell lines, opening new avenues for the evaluation of MCL-1 inhibitors in these cancers. Moreover, MCL-1 has been identified as an important biomarker: its expression, generally evidenced by immunohistochemistry, was associated with resistance to therapy and reduced progression-free survival (PFS) and overall survival (OS). As high MCL-1 expression often results in resistance to both cytotoxic agents and targeted therapies, combining MCL-1 inhibitors with these agents is generally able to overcome this resistance. We will now discuss the findings supporting the implication of MCL-1 in an array of solid and hematological tumors and review the potential combinations of MCL-1 inhibitors with preexisting therapeutic options.

### Lung cancer

Lung cancer consists of approximately 80% NSCLC and 20% small-cell lung cancer (SCLC) and remains the leading cause of cancer-related deaths. NSCLC is commonly associated with targetable kinase mutations that are less present in SCLC biology. Sixty-two percent of patients with NSCLC harbor an oncogenic driver mutation and half of them consist of a therapeutically targetable lesion [50, 51]. Mutations in KRAS and EGFR are the most common and occur in 25–30% or 11–15% of patients, respectively. Regarding treatment, the development of EGFR- tyrosine kinase inhibitors (EGFRi) resulted in some success, but the observed responses were short, due to the development of acquired resistance resulting from secondary or tertiary EGFR mutations and other mechanisms [52]. After exposure to EGFR inhibitors, EGFR-mutant NSCLC cells lose their ability to undergo apoptosis, partially due to the high expression of MCL-1 and finally become “drug-tolerant cells.” This overexpression is explained by (1) enrichment of cells with preexisting high MCL-1 expression and (2) activation of the mTORC1/eIF4E-mediated cap-dependent translation pathway that controls MCL-1 expression [52]. Combining MCL-1 and EGFR inhibitors synergistically reduced viability, induced apoptosis and prevented the development of drug-tolerant cells. This combination also reduced subcutaneous tumors in xenograft models with EGFR-mutated cell lines [52].

Micro-array and immunohistochemistry demonstrated high MCL-1 expression in NSCLC cells and its inhibition could induce NSCLC cell apoptosis [53]. In tissue samples of NSCLC, the expression of MCL-1 could be correlated to the proliferation and apoptosis index and serve as a biomarker for survival: the five-year OS of patients with MCL-1 positive staining (68.3%) was inferior as compared to the survival of those with MCL-1-negative tumors (93.1%) [54]. The expression of MCL-1 in SCLC has been studied to a lesser extent. SCLC is generally characterized by a heterogeneous BCL-2 family dependency. Seven studied SCLC cell lines displayed differential addiction to either BCL-2, BCL-xL or MCL-1 for their survival, and the predominant protein expression of BCL-2, BCL-xL or MCL-1 could be used as a surrogate marker for this selective addiction [55]. High expression of MCL-1, associated with low expression of BCL-xL and BCL-2, was recently described in a small series of SCLC biopsies [56]. The MCL-1 inhibitor S63845 reduced viability of SCLC cell lines in vitro with an IC50 of 23 to 78 nM by inducing apoptosis, while in vivo treatment of two xenograft models reduced tumor volumes to a comparable degree as cisplatin in combination with etoposide [56]. SCLCs with high BCL-xL expression were less sensitive to S63845 and its knockdown sensitized cells to MCL-1 targeting. Accordingly, the combination of navitoclax (a dual inhibitor of BCL-xL and BCL-2) and S63845 reduced the cell viability of SCLC cell lines and showed in vivo synergistic effects in S63845-resistant xenograft model [56].

Additional evidence for the attractive role of MCL-1 as a drug target in lung cancer comes from its non-apoptotic involvement in DNA repair. Targeting of MCL-1 with a small molecule inhibitor (MI-233) blocked MCL-1-mediated HR DNA repair and thereby sensitized cancer cells to treatment induced replication stress [24]. Remarkably, this strategy was effective in vivo as evidenced by strong synergism of MI-233 with either hydroxyurea or olaparib in lung cancer models. Although it needs to be proven whether these effects can be replicated with established MCL-1 inhibitors, these results demonstrate that the non-apoptotic functions of MCL-1 can be exploited to tackle key vulnerabilities of lung cancer cells.

### Breast cancer

Similar to other malignancies, breast cancer is a heterogeneous disease that encompasses distinct molecular subtypes. In the majority of mammary carcinomas, a significant portion of the malignant cells express hormone receptors for estrogen and/or progesterone. Importantly, BCL-2 is a direct transcriptional target of ERα and its expression is predominantly found in this subgroup [57].

In contrast, MCL-1 is widely expressed in all breast cancers: estrogen-receptor-positive (ER +) breast cancers, HER2-amplified and triple negative breast cancers. Alterations in regulatory pathways as well as external signals can modulate MCL-1 expression. This was elegantly illustrated by Louault et al. who showed that primary cultures of cancer-related fibroblasts (CAFs) protect breast cancer cells from chemotherapy-induced apoptosis [58]. Co-culture experiments with CAFs suggest that the increased MCL-1 mRNA expression and protein stability in cancer cells are the result of IL-6 release [58].

High levels of MCL-1, assessed by immunohistochemistry, could be correlated with high tumor grade, whereas low expression of MCL-1 was correlated with low tumor grade [59]. Accordingly, patients' survival is decreased significantly with increased expression of MCL-1 [59]. Quantification of RNA expression of the BCL-2 family proteins (BCL-2, MCL-1 and BCL-xL) in breast cancer cell lines and primary patient samples found higher MCL-1 transcripts compared to BCL-2 and BCL-xL [60]. Moreover, MCL-1 protein expression levels are linked to poor outcome, are required for breast cancer development, and targeting of MCL-1 hampers triple-negative breast cancer development in vivo [30].

Molecular profiling of tumor cells persisting after neo-adjuvant chemotherapy for triple-negative breast cancer showed amplifications of MYC(54%) and MCL-1(35%) [61]. In this study, 83% of MYC-amplified tumors exhibited co-amplification of MCL1, suggesting that MYC and MCL-1 cooperate to induce chemoresistance [61]. In a follow-up study, the authors identified an increased mitochondrial oxidative phosphorylation and the generation of reactive oxygen species as driving mechanisms of this MYC and MCL-1 induced resistance [45].

Targeted MCL-1 blockade using RNAi also increased caspase-mediated cell death in ERα + breast cancer cells, resulting in sustained growth inhibition [60]. Strikingly, ERα + breast cancer cell lines have limited sensitivity to BCL-2 inhibitors. The addition of ABT-263 to cultures of breast cancer cells results in a transient apoptosis induction, due to a rapid upregulation of MCL-1 and subsequent sequestration of BIM by MCL-1. When the mTORC1 inhibitor RAD001 was added to ER + breast cancer cells, the MCL-1 protein levels decreased and cells were able to undergo apoptosis [62]. The combination of ABT-263 with the specific MCL-1 inhibitor VU661013 was able to induce tumor cell death in vivo and a synergistic reduction in tumor growth [62].

Similar efficacy was seen when potent MCL-1 inhibitors are combined with standard therapeutics, incl. tamoxifen, trastuzumab, paclitaxel or docetaxel [63, 64]. This suggests that rationale derived combination therapies using the blockade of MCL-1 can induce cell death in all breast cancer subtypes in addition to single-agent MCL-1 inhibitor approaches in tumor clones with strong MCL-1 dependency.

### Melanoma

The introduction of immunotherapy and novel therapeutics (BRAF and MEK inhibitors) has improved the outcome of melanoma patients. However, disease relapse typically occurs due to acquired resistance through reactivation of ERK1/2 signaling [65]. ERK1/2 promotes cell survival in BRAF- or KRAS-mutant tumor cells by increasing the expression of pro-survival BCL-2 proteins and further stabilizes the MCL-1 protein [66].

Melanoma cells exhibit high levels and activity of MCL-1 protein [67, 68]. Activation of BRAF signaling results in increased expression of MCL‐1 in vitro, revealing a potential mechanism of apoptotic resistance [69]. Similar to other malignancies, knockdown of MCL-1 sensitizes melanoma cells to various treatments, including BRAF or MEK inhibitors. When different BH3-mimetics were tested on melanoma cell lines (including wild-type and mutant BRAF and NRAS lines), all cell lines were found to be resistant to the individual drugs. Combinations targeting MCL-1 plus BCL-xL or BCL-2 showed considerable synergistic killing activity that was elicited via both BAX and BAK. This synergy was also observed in 3D spheroid cultures, where BH3-mimetic combinations targeting MCL-1 plus BCL-xL were most effective at killing melanoma cells.

The power of combination approaches with BH3-mimetics in melanoma is further supported by another recent study that investigated mRNA and protein expression levels of MCL-1 and BCL-xL in different cancer models. Compared to other cancers, the MCL-1:BCL-xL mRNA and protein ratio was 2 to sixfold higher in melanoma. Because BCL-xL levels were lower in melanoma, these results indicated that MCL-1 was predominant in melanoma cell lines and patient samples [70]. The combined inhibition of ERK1/2 signaling together with MCL-1 induced synergistic apoptosis, inhibited the clonogenic survival of melanoma cells and caused regression of subcutaneous tumors in xenografts. Finally, combining MCL-1i with either BRAF or MEK inhibitors re-sensitized melanoma cells and enhanced tumor growth inhibition in vivo. While monotherapy with the BRAFi vemurafenib or the MEKi trametinib showed no anti-tumor effects in BRAF-mutant xenograft models, the combination of the MCL-1i AZD5991 with these inhibitors reduced tumor growth [70].

### Colorectal cancer

MCL-1 also protects colorectal cancer (CRC) cells from apoptosis [71]. Immunohistochemistry on tumor biopsies showed strong cytoplasmic expression of MCL-1 that could be correlated with tumor stage, lymph node metastasis and with inferior survival of CRC patients [71]. MCL-1 also protects CRC tumor cells from the apoptotic effects of targeted drugs. Indeed, several inhibitors of aberrantly activated oncogenic kinases (such as the multi-kinase inhibitor regorafenib and its analogue sorafenib) are used in chemotherapy-resistant and metastatic colorectal cancer as well as in other gastrointestinal malignancies. Early work showed that sorafenib acts by inhibiting the initiation factor eIF4E and subsequently decreasing MCL-1 protein levels [72]. Constitutive overexpression of MCL-1 in CRC cells significantly inhibits sorafenib-induced apoptosis, whereas MCL-1 down-regulation by RNA interference enhances sorafenib-induced apoptosis [73].

Lin Zhang et al. extensively dissected the mechanisms of resistance that CRC cells acquire toward regorafenib. They first identified that inactivating mutations in the previously described *FBW7* gene contribute to resistance to these multi-kinase inhibitors [74]. Analysis of BCL-2 family members revealed a dose- and time-dependent depletion of MCL-1 in regorafenib-treated wild-type CRC cells. Clones with hotspot mutations in the *FBW7* gene do not show this MCL-1 depletion and thus survive and become dominant upon chronic drug exposure. MCL-1 knockout in CRC cells or the addition of an MCL-1 inhibitor confirmed the critical role of MCL-1 in apoptosis. In both experiments, the sensitivity toward regorafenib was restored in each of the resistant cell lines [74].

In a follow-up study, regorafenib was found to inhibit upstream ERK signaling and to activate GSK3β-mediated MCL-1 phosphorylation [75]. In line with this, BRAF^V600^^E^ mutations in CRC cells were associated with MCL-1 upregulation, stabilization via phosphorylation at Thr163 and chemoresistance [76, 77]. This vicious cycle can be targeted with trametinib which was shown to enhance FBW7-mediated MCL-1 degradation (via the phosphorylation of the PEST sequence) in CRC cells [78]. Mutations within the PEST sequence blocked this phosphorylation process and resulted in increased MCL-1 half-life, increased sequestration of PUMA and resistance to regorafenib [79]. Interestingly, the MCL-1 inhibitors S63845, AZD5991 or AMG176 restored the sensitivity to regorafenib in both CRC cells with intrinsic *FBW7* mutations or acquired mutations in MCL-1 in different in vitro and in vivo experiments [79]. In resistant CRC cells, immunoprecipitation studies found a strong binding of MCL-1 to the proapoptotic BH3-only protein PUMA that was disrupted after treatment with the MCL-1 inhibitor S63845. These results confirm that two events are necessary for initiating apoptotic response to regorafenib, including early MCL-1 degradation and later PUMA induction.

## Hematological Malignancies

During lymphoid and myeloid differentiation, the expression of BCL-2 family proteins is highly variable. During B-cell development, MCL-1 is implicated in every differentiation step: it is essential for germinal centre formation and maintenance, memory B-cell development and survival of plasma cells [80]. In contrast, BCL-2 is important for naïve and memory B-cells, but is downregulated in the germinal center. Most of the B-cell malignancies mimic the BCL-2 family expression profile of their normal counterparts [80]. Further evidence that the cell of origin plays an important role in anti-apoptotic dependencies and BH3 mimetic sensitivity comes from studies in AML and T-ALL [81, 82]. The observed responses to a combination therapy of venetoclax and azacitidine for AML correlated closely with the developmental stage, where phenotypically primitive AML was sensitive, but monocytic AML was more resistant [82]. The latter had a distinct transcriptomic profile with loss of BCL-2 and gains of MCL-1 for their survival. Similarly, the maturation stage of T-ALL determined its anti-apoptotic dependency and sensitivity to BH3-mimetics. Mitochondrial BH3 profiling showed that most of T-ALL cells were dependent upon BCL-XL, except an early T-cell progenitor subgroup that was dependent on BCL-2 [81].

### Multiple myeloma

MM is thought to represent a prime example of an MCL-1-dependent malignancy, which might arise from the crucial role of MCL-1 for the survival of normal plasma cells [83]. This is underlined by the impressive impact of MCL-1 targeting strategies. Initial preclinical efforts demonstrated that only MCL-1, but not BCL-2 or BCL-xL targeting anti-sense oligonucleotides are capable to impair MM cell survival [84, 85]. A finding that is supported with today’s clinical-grade BH3-mimetics. Although promising results were obtained in a subset of MM patients treated with venetoclax, this activity is restricted to tumor clones with high BCL-2:MCL-1 and BCL-2:BCL-xL expression ratios as well as reduced electron transport chain activity [86, 87]. This venetoclax-sensitive phenotype is enriched in MM patients with tumor clones carrying a t(11;14) [88]. In contrast, most other MM subgroups rely on MCL-1 and are thus insensitive to single-agent BCL-2 or BCL-xL inhibition as evidenced by pharmacological inhibition and CRISPR screens [7].

The analysis of BCL-2 family members in primary MM cells strengthens the predominant role of MCL-1. Increased MCL-1 expression discriminates MM cells from “normal” plasma cells and is linked to poor outcome [89]. Interestingly, MCL-1 dependency is further enhanced during disease progression. Gomez-Bougie et al. observed a 36% increase of MCL-1-dependent MM cells from diagnosis to relapse based on BH3 mimetic activity results [90]. Accordingly, BH3 profiling and interaction analyses of pro- and anti-apoptotic BCL-2 family members (e.g., MCL-1/BCL-xL/BCL-2 binding to Bim) predict the efficacy of BH3-mimetics in MM [6, 91]. Conflicting results exist in relation to the impact of amplification of 1q21 and MCL-1 inhibitor sensitivity [92, 93].

MCL-1 in myeloma is regulated at both the transcriptional and the functional levels. The major MM survival factor interleukin 6 (IL-6) and interferon alpha (IFNa) were shown to promote MCL-1 protein expression in a STAT3-dependent manner, but not those of other BCL-2 family members [94]. Alternative extracellular triggers of increased MCL-1 expression include VEGF, BAFF, APRIL and sphingosine-1-phosphate. Gain of the chromosomal region 1q21 and deregulation of MCL-1 targeting miRNAs were likewise shown to contribute to aberrant MCL-1 expression in MM cells [93]. More recently, IL-6-induced MCL-1 dependency in MM was linked to its concurrent reliance on bone marrow stromal support. Gupta et al. were able to demonstrate that IL-6 alters the phosphorylation status of BIM in myeloma cells and thereby promotes the formation of MCL-1:BIM complexes (which outcompetes BCL-2 or BCL-xL binding to BIM) at least in part in a MEK signaling-dependent manner [95]. Noteworthy, this IL-6-induced functional switch in favor of MCL-1 dependency is independent of changes in the MCL-1 expression levels. MM cells—in turn—also alter their surrounding environment to stimulate the formation, functionality and survival of myeloid suppressor cells via upregulation of MCL-1 through STAT3/STAT1 activation [96]. Consequently, MCL-1 also appears as an attractive drug target to overcome the immunosuppressive features within the myeloma niche.

Finally, MCL-1 plays not only a role in myeloma survival and drug resistance but also in the efficacy of established drugs. The presence of a short, cleaved form of MCL-1 was implicated in both melphalan and bortezomib induced apoptosis in MM cells. Melphalan exposure induces the dissociation of MCL-1 (but not BCL-2) from BIM and the caspase dependent cleavage of MCL-1 into its pro-apoptotic form. This leads to the release of BIM and the exertion of its pro-apoptotic function [97]. Proteasome inhibitors were likewise shown to induce caspase dependent MCL-1 cleavage. This went in line with a prominent upregulation of NOXA, increased presence of MCL-1:NOXA complexes, release of BIM from MCL-1 and induction of apoptosis [98, 99]. In addition, nuclear translocation of MCL-1 was reported to induce apoptosis via c-Jun upregulation [100]. The short half-life of MCL-1 also affects the potency of mitotic inhibitors in myeloma. Mitotic arrest leads to a rapid loss of MCL-1 and induction of apoptosis in myeloma cells, whereas MM cells with sustained expression of anti-apoptotic molecules (e.g., BCL-xL) or the capability to evade via mitotic slippage are less prone to mitotic blockers [101]. Overall, these data demonstrate a key role for MCL-1 in myeloma cell survival and for the efficacy of established backbone therapeutics in MM.

### Non-Hodgkin lymphoma

The importance of MCL-1 as a potential therapeutic target in lymphoma is underscored by the limited clinical success of the BCL-2-inhibitor venetoclax, which achieved an overall response rate of 44% in a phase 1 clinical trial in Non-Hodgkin lymphoma (NHL) (MCL, 75%; FL, 38%; DLBCL, 18%) [102] and 26% in a real-world off-label NHL patient cohort [103]. MCL-1 has been identified as a resistance factor for venetoclax in multiple studies [104–106], indicating that survival of lymphoma cells can be mediated by both BCL-2 and MCL-1.

Screening of a large tumor cell line panel comprising 952 cancer cell lines, across different malignancies for their sensitivity to a precursor of AMG-176 (AM-8621), identified B-cell lymphoma as most dependent on MCL-1, next to MM [107]. Of note, this screen also included venetoclax and thus allowed for a direct comparison of BCL-2 and MCL-1 as therapeutic targets. B-cell lymphoma comprises Hodgkin lymphoma as well as NHL, with DLBCL being the most aggressive type of NHL. Among the DLBCL cell lines included in this panel, several cell lines displayed a dependency on MCL-1, BCL-2, both, or neither BCL-2 nor MCL-1, highlighting the heterogeneity of this disease in regard to the BCL-2 family [107]. This heterogeneity was also observed in a study investigating the function of the different anti-apoptotic BCL-2 proteins in DLBCL by comparing the efficacy of different BH3-mimetics [108]. Of note, when comparing the mRNA expression of the main anti-apoptotic BCL-2 proteins in a large cohort of primary DLBCL tissues, expression of MCL-1 was higher and more homogenously high than expression of BCL-2 or BCL-xL. These data demonstrate that although MCL-1 is expressed rather uniformly at high levels at the mRNA and protein level, only a subset of cells are dependent on MCL-1 and sensitive to MCL-1-inhibition. Sensitivity to MCL-1-inhibitors is influenced by the expression of other anti-apoptotic BCL-2 proteins, but also by the interaction pattern of MCL-1 with pro-apoptotic BH3-domain containing proteins [108]. Of note, two independent studies have identified high expression of BCL-xL as the most predictive factor for resistance to MCL-1-inhibitors, indicating that BCL-xL (but not BCL-2) can functionally compensate for the inhibition of MCL-1 [107, 109]. However, these correlation analyses have not yet been performed in larger panels of lymphoma cells, and a comparison across different tumor types in particular when including solid tumors may underestimate a potential role of BCL-2.

Primary effusion lymphoma is a rare subtype of DLBCL that currently has limited treatment options and occurs in individuals with Kaposi’s sarcoma-associated herpesvirus. A recent CRISPR/Cas9 knockout screen identified MCL-1 as a key anti-apoptotic driver in primary effusion lymphoma, highlighting the potential of MCL-1-inhibitors as novel therapeutic option in this aggressive malignancy [110].

Early clinical data as well as preclinical data indicate that also in mantle cell lymphoma, which is largely characterized by high expression of BCL-2 and showed a more promising clinical response to venetoclax than other subtypes of B-cell lymphoma [102], MCL-1 may be a valuable therapeutic target. This may be particularly the case in the context of acquired resistance to venetoclax, which has been described to be associated with a shift toward MCL-1-dependency [111]. Therefore, inhibition of MCL-1 may be a particularly promising strategy in patients that relapse after venetoclax treatment. Preclinical data indicate a high level of synergy between MCL-1- and BCL-2-inhibition, and clinical trials investigating the combination of BCL-2- and MCL-1-inhibition are currently ongoing. However, this treatment is anticipated to be associated with increased toxicity on normal blood cells, and it remains to be determined under which conditions a safe therapeutic window can be achieved.

Interestingly, early observations indicate that c-MYC-driven Burkitt lymphoma may be particularly dependent on MCL-1, with 7 out of 7 tested cell lines responding to nanomolar concentrations of S63845 [109]. This link between c-MYC-driven cancers and a dependency on MCL-1 was confirmed by genetic studies, where heterozygous deletion of MCL-1 resulted in diminished growth of c-MYC-driven lymphomas [112].

### Chronic lymphocytic leukemia

Inefficient apoptosis is a hallmark of chronic lymphocytic leukemia (CLL). Overexpression of the anti-apoptotic protein BCL-2 is considered primarily responsible for the increased apoptosis resistance and prolonged survival of CLL B cells and explains the rationale of using a specific BCL-2 inhibitor (venetoclax) in CLL. The approval and current use of venetoclax in the treatment of CLL generated interest in studying and targeting the other BCL-2 family proteins. Early work showed high mRNA and protein levels of MCL-1 in CLL cells [113]. These levels could be correlated to resistance to chemotherapeutic agents and monoclonal antibody therapy with rituximab [113, 114]. In this context, stromal-driven drug resistance was shown to be a mediator of MCL-1-associated chemo-resistance. Co-culture of CLL cells with stromal cells induces MCL-1 mRNA and protein expression in concert with AKT, ERK and GSK3β phosphorylation, regulating MCL-1 expression and protein [115].

The B-cell receptor is a key signaling molecule that triggers pathways that can induce B-cell proliferation, survival, differentiation, anergy and apoptosis. Sustained engagement of the BCR induces MCL-1 through activation of PI3 kinase and results in resistance toward chemotherapy [116]. Its downstream signaling can be blocked with the Bruton tyrosine kinase-inhibitor ibrutinib. Activation of the BCR protected CLL cells from venetoclax-induced apoptosis through induction of MCL-1. This dependency on MCL-1 was confirmed by siRNA knockdown of MCL-1 that completely reversed resistance of BCR-activated CLL cells to venetoclax [117]. High levels of MCL-1 were shown to inversely correlate with treatment response in the phase 1 study of ABT-263 in CLL [118]. Finally, different signaling inhibitors (BTK, PI3 kinase and SYK kinase inhibitors) could overcome BCR-mediated venetoclax resistance [117].

Targeting MCL-1 directly with selective inhibitors, or indirectly with agents that cause downregulation of MCL-1 as part of their mechanism of action, proved to be efficient in preclinical models of CLL [119, 120]. More recently, new MCL-1 inhibitors were preclinically tested in CLL. One of them, AMG 176, was able to induce cell death at nanomolar concentrations; this effect was seen in monocultures of primary CLL cells and in co-cultures with stromal cells. Only minor differences were observed in different cytogenetic subtypes of CLL. AMG 176 induced CLL cell death via the mitochondrial pathway with an increase of BAX and BAK in the mitochondrial protein fraction, indicating oligomerization of these proteins. Finally, a synergistic effect was observed between venetoclax and AMG-176 [121].

### Acute myeloid leukemia

The approval of venetoclax plus hypomethylating agent or low dose cytarabine for acute myeloid leukemia (AML) has brought forth a new treatment landscape for this disease [122, 123]. Nevertheless, many patients will not respond or will eventually relapse. While the mechanisms of resistance to venetoclax in AML may vary, resistance is primarily believed to be driven by a shift in dependency to other anti-apoptotic factors such as MCL-1. Recent investigations, however, have highlighted enrichment of clones driven by enhanced FLT3 or RAS signaling, or biallelic loss of TP53 in AML patients who have acquired or had primary resistance to venetoclax [124]. High levels of MCL-1 expression were identified in AML clones with internal tandem duplications (ITD) of FLT3. Upregulation of MCL-1 in FLT3-ITD clones is driven by constitutive STAT3 and/or STAT5 signaling and correlates with functional MCL-1 dependency as evidenced by cell death induction in response to MCL-1 silencing [125, 126].

Hierarchical analysis of major pro-survival proteins in murine and human AML likewise demonstrated that MCL-1 is the major anti-apoptotic protein in AML. Glaser et al. showed that Mcl-1 was critical for the sustained survival and expansion of mouse as well as human AML, whereas Bcl-xL, Bcl-w or Bcl-2 only play minor prosurvival roles in these cancers [33]. This observation was independent of the mutational status of the tested cell lines, and only apoptosis refractory cells (i.e., cells harboring BAX/BAK downregulation or BAK mutation) remained viable in the absence of MCL-1 [33]. In accordance with these findings, MCL-1 upregulation is frequently found at disease recurrence [127] and high levels of MCL-1 are linked to poor outcome.[128]. In contrast to this, mitochondrial priming measured by BH3 mimetic peptides showed primary AML cells from both refractory and sensitive patients responded more to the BAD BH3 peptide, which suggested a specific dependence on BCL-2 or BCL-w for these cells' survival. These results, coming from conditional knock-out and from mitochondrial priming studies, indicate that there are different subgroups in AML with reliance on different anti-apoptotic proteins.

More recent data provided important insights into the dynamics of BCL-2 family members in different subgroups of AML. This revealed a heterogeneous expression profile of BCL-2 and MCL-1, with clear upregulation of the latter in monocytic AML. Intriguingly, monocytic AML is venetoclax resistant which relies on its dependency on MCL-1 for oxidative phosphorylation and survival [82, 129].

This culminative evidence highlights the essential role of MCL-1 in the development and sustained growth of AML and thus places AML at the center of the ongoing implementation studies of MCL-1 targeting therapeutics.

The clinical exploration of MCL-1 inhibitors in AML is further supported by impressive preclinical in vitro and in vivo results with modern MCL-1 targeting BH3-mimetics. AML together with myeloma was revealed as the prime indication for these drugs (cfr Table 2). Also, side by side comparisons of BH3-mimetics targeting MCL-1, BCL-2 or BCL-xL underlined the role of MCL-1 as key target in AML [130]. MCL-1 inhibitors rapidly induce apoptosis in AML cells in a BAK dependent manner. This established activity profile together with its role in drug resistance makes it an attractive target for combination therapy approaches. For instance, venetoclax induces apoptosis in a BAX-dependent manner [130]. Therefore, several clinical trials are currently exploring the impact of simultaneous BCL-2 and MCL-1 inhibition in AML. These efforts are supported by encouraging preclinical data demonstrating the feasibility and potent activity of this combination approach [131]. Moreover, combined BCL-2 + MCL-1 inhibition outperformed the activity of standard drugs.

Regarding the treatment of recurrent disease, MCL-1 inhibition was shown to be an effective approach to (re)sensitize resistant AML cells to prior drugs. As expected, MCL-1 inhibition is able to reverse venetoclax or ABT-737 resistance [107, 132, 133]. In addition, MCL-1 inhibition is capable to sensitize AML cells to multiple other drugs. Suppression of MCL-1 in FLT3-ITD AML cells significantly sensitized cells to cytarabine and daunorubicin. More recent data also pointed to superior activity of BET protein inhibitors in combination with either BCL-2 or MCL-1 targeting BH3-mimetics [134]. Finally, the association between MCL-1 expression or dependency and genetic events in AML patients offers excellent strategies for precision medicine approaches. Besides FLT3-ITD, PTPN11 mutations are closely linked to MCL-1 upregulation, venetoclax resistance and metabolic reprogramming toward increased oxidative phosphorylation and glycolysis. These tumor clones are highly sensitive to MCL-1 inhibition, which is linked to decreased oxidative phosphorylation, thus overcoming unmet medical needs in AML [135, 136].

## Targeting of MCL-1 with small molecule inhibitors

The poor selectivity and low affinity of the first inhibitors and the reduced bioavailability of others (e.g., A-1210477) delayed the clinical development of potent MCL-1 inhibitors. Specific targeting of MCL-1 is particularly challenging due to its large, surface-exposed hydrophobic BH3 binding groove [137, 138]. Some of the putative MCL-1 inhibitors (Gossypol and its derivatives), which demonstrated high efficacy in preclinical studies, showed various off-target effects not associated with the direct inhibition of MCL-1 (e.g., upregulation of NOXA) [139]. The indirect targeting of MCL-1 was also exploited as key mechanism of action of alternative drug classes such as CDK9 inhibitors or deubiquitination inhibitors [140, 141]. However, during the evolution of MCL-1 inhibitors, their affinities improved from micromolar to subnanomolar which finally enabled the design and synthesis of highly potent and specific clinical-grade MCL-1 inhibitors (Table 1). Up to date, three published compounds moved into clinical trials.

**Table 1:** Pharmacological characteristics of the main MCL-1 inhibitors

Compound	Affinity	MCL1 stabilization	Activity correlates with MCL-1 expression	Inverse correlation between activity and Bcl-xL expression	BAK dependent activity	Activity in solid tumors?
S63845	K_i_ < 1.2 nM	Yes	No	Yes	Yes	Few: NSCLC, breast cancer, melanoma
AMG 176	K_i_ = 0.06 nM	Yes	No	Yes	Yes	Breast cancer
AZD5991	K_i_ = 0.2 nM	Yes	No	Yes	Yes	n/a
VU661013	K_i_ = 97 ± 30 pM	n/a	no	-(not at protein level)	n/a	n/a
Compound 42	K_i_ = 0.03 nM	n/a	n/a	Yes	Probably (> 10-fold shift in IC50 in BAX/BAK KO cells)	TNBC, but lower activity as compared to hematological tumors (promising activity in chemotherapy combination approaches)
b-carboline copper(II) complexes	K_i_ = 1.2–96.4 nM	n/a	n/a	n/a	Yes	NSCLC

The first published compound of these pioneering activities was S63845 [109]. The lead compound for S63845 was derived from thienopyrimidine amino acids and further developed via an NMR-based fragment screen in combination with structure-guided drug design [142]. The compound binds to conserved Arg263 and the P2 plus P4 hydrophobic pockets of the MCL-1 BH3 binding groove with a K_D_ of 0.19 nM and K_i_ < 1.2 nM. Concurrent binding to either BCL-2 or BCL-xL was not observed (K_i_ > 10 000 nM). As expected, S63845 displayed impressive potency at low nanomolar concentrations in preclinical in vitro and in vivo models of hematological malignancies, including MM, AML, CML and c-MYC-driven Burkitt lymphoma. S63845 treatment (25 mg/kg) led to a complete tumor regression at day 100 post-treatment in xenograft models of MM and a 70% cure rate in immune-competent Eµ-MYC mouse lymphomas, respectively [109]. Concerns about potential dose-limiting adverse events due to the six-fold lower affinity of S63845 for murine vs. human MCL-1 were retracted by recent data obtained in a humanized MCL-1 mouse model. S63845 treatment at the maximum tolerated dose of 12.5 mg per kg cured 60% of humanized Eµ-MYC lymphomas with only a transient reduction of B cells and red blood cells [143]. This underlines the presence of a therapeutic window which is currently evaluated with the S63845-related compound S64315/MIK665 in ongoing clinical studies. The antitumor activity of S64315/MIK665 was studied in different in vitro and in vivo models of hematological malignancies (AML, MM and DLBCL). In vitro, a strong synergy was observed when S64315/MIK665 was combined with specific BCL-2 inhibitors. This synergy was confirmed in vivo where durable responses were seen in xenograft models [144].

Following the path of S63845, the highly potent and specific MCL-1 inhibitors AMG 176 and AZD5991 were disclosed in 2018 [107, 145]. AMG 176 represents the first-in-class orally bioavailable MCL-1 inhibitor. AMG 176 was obtained using structure-guided drug design approaches and conformational restriction to optimize its properties (K_i_ = 0.06 nM). On-target activity was verified in preclinical models and large cell line screenings (n = 952) revealed hematological malignancies (MM, AML, B-cell lymphoma, Burkitt lymphoma, ALL) as the indications of highest relevance. The potency of AMG 176 was verified in MM xenograft models and an orthotopic model of AML at doses of 30 mg/kg and 60 mg/kg, respectively [107]. Similar to S63845, AMG 176’s affinity for murine MCL-1 is significantly reduced as compared to human MCL-1 (K_i_ = 0.044 µM). Thus, Caenepeel et al. likewise evaluated AMG 176 in a humanized MCL-1 knock-in mouse model demonstrating dose-dependent decreases in B cells, monocytes and neutrophils. However, no overt systemic toxicity was observed at doses of 30 mg/kg or 60 mg/kg and also the combination treatment with venetoclax was well tolerated [107]. AMG 397, derived from AMG 176, is more potent and has an improved pharmacokinetic profile [146]. Cell lines from hematologic malignancies including AML, multiple myeloma and DLBCL exhibited greatest sensitivity to AMG 397 in a large tumor cell line profiling screen. Administration of AMG397 to MM or AML bearing mice resulted in significant tumor regressions and in synergistic activity when combined with venetoclax [146].

AZD5991 is the fourth published MCL-1 inhibitor that is currently undergoing clinical testing. AZD5991 was obtained by optimization of previously reported indole-2-carboxylic acids. Structure-guided design led to the synthesis of a highly potent and selective compound (K_i_ = 0.2 nM) with no overt binding affinity for BCL-2 or BCL-xL (K_i_ = 6.8 and 18 µM, respectively). Similar to other MCL-1 inhibitors, AZD5991 demonstrated strong selectivity for hematological cancer cell lines, whereas only subsets of solid tumors were characterized as sensitive (NSCLC, breast cancer). In MM xenograft models, AZD5991 at 100 mg per kg resulted in complete tumor regression. 88% tumor regression was noted at sub-optimal concentrations (30 mg per kg) in combination with bortezomib. Accordingly, 71% of tested primary MM cells (n = 48) were found to be highly AZD5991 sensitive (EC50 < 100 nM). Similar efficacy was observed in murine and rat AML models [145].

Several additional compounds that were reported to specifically target MCL-1 have been disclosed during the last two years. VU661013 was developed by the group of Stephen Fesik and possesses a remarkable target affinity (K_i_ of 97 ± 30 pM) with no sign of cross-binding to BCL-2 (K_i_ = 0.73 µM) or BCL-xL (K_i_ > 40 µM) [147]. VU661013 demonstrated potent in vitro and in vivo activity in models of AML as well as patient-derived AML cells and xenograft models. Moreover, VU661013 showed strong synergy with venetoclax and its activity correlated with BH3 profiling which confirms the applicability of this assay for biomarker studies. Interestingly, residual AML cells isolated from VU661013 in vivo studies displayed MCL-1 inhibitor resistance with a concurrent increase in the sensitivity to venetoclax [147]. Ongoing efforts in the Fesik lab recently led to the synthesis of compound 42. The development of this substance used structure-based design and several rounds of optimization starting from tricyclic diazepinones [148]. Compound 42 halted tumor growth and was well tolerated at doses up to 100 mg per kg in xenograft models of MM and TNBC. Although compound 42 activity was limited in the latter, strong synergism was observed in vivo in combination with docetaxol or doxorubicin which is of great promise for the introduction of MCL-1 inhibitors outside the range of hematological malignancies.

In addition to the mentioned compounds, several alternative strategies are currently exploited to obtain novel clinical-grade MCL-1 inhibitors. For instance, b-carboline copper(II) complexes were described as MCL-1 inhibitors that act in a BAX/BAK dependent manner and show potent anti-tumor activity in preclinical cancer models that was even superior as compared to the activity of AZD5991 (57% vs 23% tumor growth inhibition at 10 mg per kg in NCI-H460 tumor cell xenografts) [149]. These data can be used to build on for the further development of more potent and specific metal-based MCL-1 inhibitor structures. Also, proteolysis-targeting chimeras (PROTACs) represent a promising approach to target MCL-1. PROTACs are engineered bifunctional molecules composed of a target-binding ligand joined via a linker to an effector ligand that binds to E3 ubiquitin ligase to trigger proteasomal degradation of the target protein [150]. In 2019, two MCL-1 PROTACs were reported. The first one linked a moiety of the MCL-1 inhibitor A1210477 to 4-hydroxythalidomide, a thalidomide metabolite, that binds to the cereblon (CRBN) pole [151]. This PROTAC had a K_D_ of 30 nM with MCL-1 and directly degraded MCL-1 via the E3 ligase and cereblon-directed proteasome–ubiquitin pathway. The second PROTAC combined another MCL-1 binding compound and the cereblon–ligand pomalidomide. This compound selectively induced a 70% decrease in MCL-1 levels of treated HELA cells lines at 1 μM and progressively downregulated it to less than 10% at 10 μM [152].

Taken together, different drug development strategies led to the recent development and clinical translation of potent MCL-1 inhibitors. These compounds are characterized by their impressive potency, on-target activity (i.e., BAK dependent induction of apoptosis), a stabilizing effect on MCL-1 protein at least in non-sensitive cells, an inverse correlation between activity and BCL-xL expression levels as well as their preferential activity in hematological malignancies. These characteristics are shared among the mentioned MCL-1 inhibitors, suggesting that they can be used to define this novel drug class. (Table 1) [147].

## Clinical development

Soon after their disclosure, the first dose-finding studies were started by Servier/Vervalis, Amgen and Astra-Zeneca (cfr Table 2). Instead of S63845, S64315/MIK665 was used in monotherapy or in combination with venetoclax for phase I trials in AML/MDS and myeloma.

**Table 2:** Current clinical trials with MCL-1 inhibitors

Compound	Company	Trial Nr	Study	Administration	Indication
S64315	Servier	NCT02979366	Phase I, monotherapy	IV	AML/MDS
S64315	Servier	NCT03672695	Phase I, combination with venetoclax	IV	AML/MDS
MIK665	Novartis	NCT02992483	Phase I, monotherapy	IV	MM/Lymphoma
ABBV-467	Abbvie	NCT04178902	Phase I, monotherapy	IV	MM
AZD5991	Astra-Zenaca	NCT03218683	Phase I, monotherapy and combination	IV	Refractory Hematological malignancies
PRT1419	Prelude Therapeutics	NCT04543305	Phase I, monotherapy	Oral	Refractory Hematological malignancies
AMG397	Amgen	NCT03465540	Phase I, monotherapy	Oral	MM/AML/NHL
AMG176	Amgen	NCT02675452	Phase I, Monotherapy	IV	MM/AML
AMG176	Amgen	NCT03797261	Phase I, combination with venetoclax	IV	AML/Lymphoma

These first pioneering studies were restricted to hematological malignances, which is not surprising given their dependence on MCL-1. Preliminary results on the first 26 patients with relapsed MM, treated with increasing doses of AMG-176, were recently disclosed [153]. The maximum tolerable dose was not reached. The side-effects were mostly hematological (neutropenia and anemia) and gastro-intestinal (nausea and diarrhea). An anti-tumor effect was seen in 11 patients with 1 complete remission, 2 partial responses and 8 patients with stable disease. A first alert concerning on-target/off-tumor toxicity came from the AMG-397 trial that was put on hold because of cardiac side effects. Because MCL-1 is implicated in normal cardiac myocyte functioning, these side effects are probably directly related to the on-target activity of these compounds and may limit the therapeutic window of MCL-1 inhibitors.

In addition to the compounds described in the previous sections, two undisclosed compounds are being used in two recent studies. Abbvie, who has a long tradition in BCL-2 inhibiting compounds, is testing ABBV-467 in monotherapy for multiple myeloma patients. Prelude Therapeutics is starting a phase I clinical trial with an undisclosed MCL-1 inhibitor PRT1419. This compound showed selective inhibition of MCL-1, and its oral administration to tumor-bearing mice resulted in tumor regression. This company patented two classes of MCL-1 inhibitors: one based on chemically modified esters of propionic acid (WO2020123994) and a second one based on spiro-sulfonamide derivatives (WO2020097577).

**Fig. 4:**
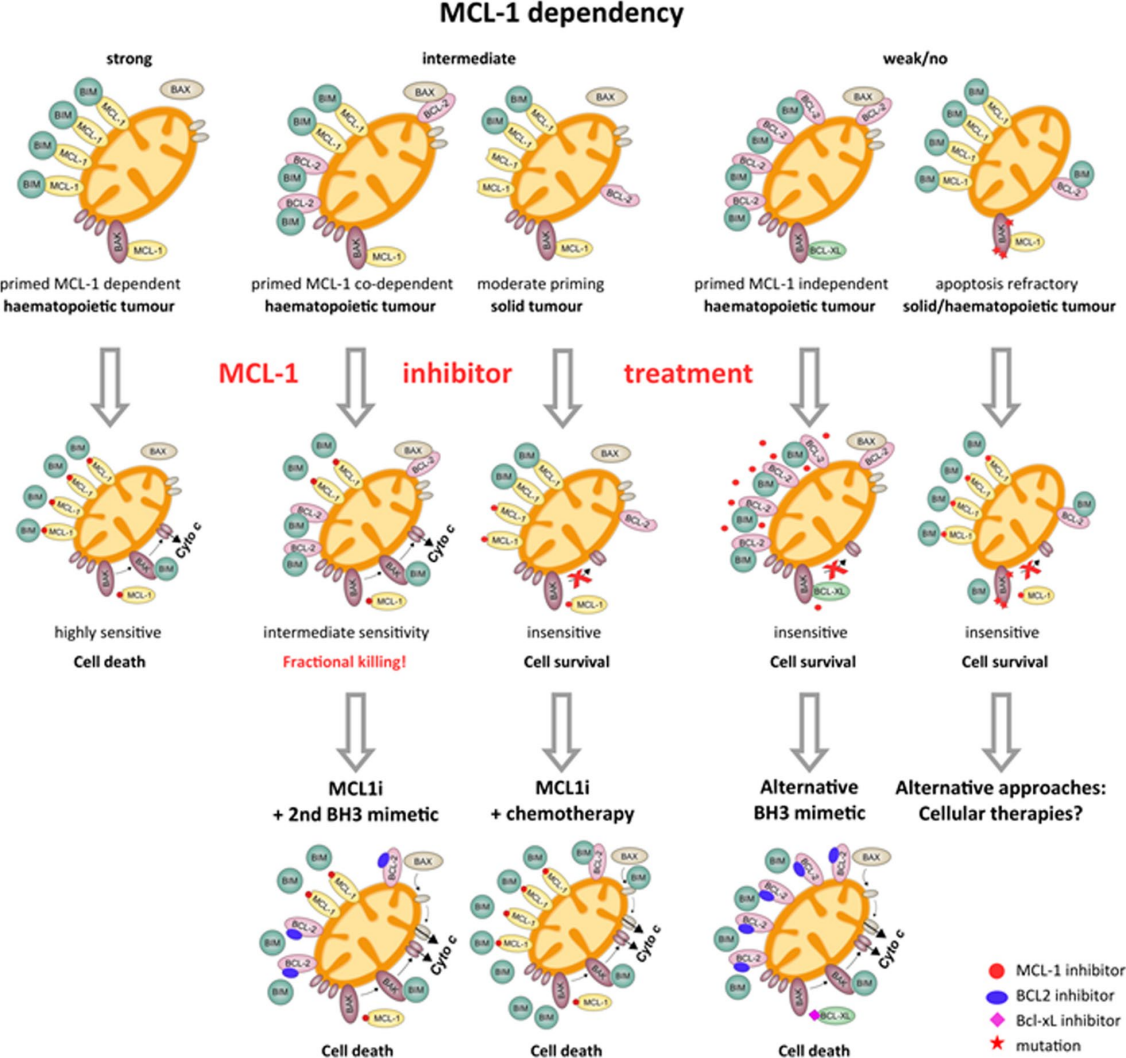
Heterogeneous BCL-2 family dependency profiles direct the application strategies for MCL-1 inhibitors in cancer. The prospects of MCL-1 inhibitors are closely linked to the mitochondrial priming status of tumor cells. In principle, cancerous cells are primed for apoptosis as compared to healthy cells due to the, e.g., oncogene associated upregulation of the apoptosis machinery. This leads to a tight balance between pro-survival and pro-apoptotic proteins. Hematopoietic cancer cells are typically more primed than solid tumors due to the interconnectedness between age, tissue location and priming status (Sarosiek et al. 2017, Cancer Cell). Consequently, MCL-1 inhibitors have great potential as single-agent therapies in primed, strongly MCL-1-dependent cancers (left panel). In tumors with either moderate priming status (e.g., solid tumors) or primed, but MCL-1 co-dependent status, combination therapies are of maximal benefit (mid panel). In primed co-dependent cells, the addition of alternative BH3-mimetics (e.g., venetoclax) is a promising strategy. In solid tumors with moderate priming, chemotherapies can lead to the upregulation of pro-apoptotic BH3-only proteins (e.g., BIM) and thus prime tumor cells for apoptosis, which can be exploited by the addition of MCL-1 inhibitors. Finally, MCL-1 inhibitors are not effective in tumor cells with weak/no MCL-1 dependency (right panel). These cells can be either targeted by alternative BH3-mimetics (e.g., primed BCL-2 dependent cells) or alternative therapy classes to tackle apoptosis refractory cells. The latter are characterized by a loss of effector molecules (BAK, BAX), downregulation of pro-apoptotic BH3-only proteins (e.g., BIM) and/or the occurrence of inactivating mutations (e.g., BAK mutations). The precise targeting of apoptosis refractory cells therefore remains a major challenge irrespective of the availability of BH3-mimetics. This view on the application strategies of MCL-1 inhibitors is based on the work by Kristopher Sarosiek and Anthony Letai, recently reviewed by Singh et al. (Nat Rev Mol Cell Biol, 2019)

In spite of the drawback with AMG-397, the ongoing clinical exploration of the the additional compounds raises hope that at least one of these molecules will present a favorable safety profile and clinical efficacy. At that moment, one can imagine extrapolation toward solid tumors where these compounds could be combined with either chemotherapeutic treatments or targeted treatments. More stratified personalized treatment approaches could be proposed based on BH3 profiling to define the priming status of cancer cells as well as dependency toward one of the anti-apoptotic BCL-2 family proteins [154]. BH3 profiling uses synthetic peptides, often derived from the BH3 domain of the pro-apoptotic BCL-2 family members, to dissect the functional relevance of each BCL-2 family member in a cell’s apoptosis machinery [154, 155]. It should be stressed that conventional mRNA expression is insufficient to predict the dependency on anti-apoptotic proteins or the response to specific inhibitors (as illustrated in Table 1). Functional assays (e.g., mitochondrial profiling or CRISPR/Cas9 screens) are more adequate to study the implications of distinct BCL-2 family members for tumor cell survival. Combining this BH3 profiling with clinically active compounds targeting MCL-1 or BCL-2 would thus open the door to biomarker-driven medicine (Fig. 4).

## Conclusion

MCL-1 contributes to tumor cell survival by interfering with the apoptotic machinery. Previous studies showed that hematological malignancies are highly dependent on MCL-1. Highly potent and specific inhibitors have been developed and entered into clinical trials for refractory hematological malignancies. The first results show some promising anti-tumor effects, but alerted also for potential cardiac toxicity. Combination of MCL-1 inhibitors with other targeted treatments or chemotherapeutic regimes will allow to overcome intrinsic apoptosis-resistance and to include them in biomarker-driven treatments. The latter should be based on functional assessments of BCL-2 family dependencies, because RNA or protein expression is not predictive for response to MCL-1-inhibitors. With the clinical development and success of the BCL-2 inhibitor venetoclax in mind, MCL-1 inhibitors may become the next novel class of anti-tumor agents with considerable potential across different malignancies.

## Data Availability

Not applicable.

## Abbreviations

AKT: AKT serine/threonine kinase 1
AML: Acute myeloid leukemia
APRIL (TNFSF13)): A proliferation-inducing ligand
BAD: BCL-2-associated agonist of cell death
BAFF (TNFSF13B): B-cell-activating factor
BAK (BAK1): BCL-2 antagonist/killer 1
BAX: BCL-2-associated X
Bcl-2: B-cell lymphoma 2
BCL-W (BCL2L2): Bcl-2-like protein 2
BCL-xL: B-cell lymphoma-extra large
BCR: B cell receptor
BFL1 (BCL2A1): Bcl-2-related protein A1
BH1,3,4: Bcl-2 homology 1,3,4
BID: BH3 interacting domain death agonist
BIM (BCL2L11): BCL-2 Like 11
BMF: Bcl-2 modifying factor
BOK: BCL-2-related ovarian killer
BRAF: B-raf proto-oncogene, serine/threonine kinase
CAFs: Cancer-related fibroblasts
Cas9: CRISPR associated protein 9
CDK9: Cyclin-dependent kinase 9
CLL: Chronic lymphocytic leukemia
CRC: Colorectal cancer
CRISPR: Clustered regularly interspaced short palindromic repeats
DLBCL: Diffuse large B-cell lymphoma
DRP-1: Dynamin-related protein 1
EGFR: Epidermal growth factor receptor
eIF4E: Eukaryotic translation initiation factor 4E
ER +: Estrogen-receptor-positive
ERK: Extracellular signal-regulated kinase 2
Erα (ESR1): Estrogen receptor 1
FBW7: F-Box and wd repeat domain containing 7
FLT3: Fms-related receptor tyrosine kinase 3
FNRS: Fonds national de la recherche scientifique
GSK3β: Glycogen synthase kinase 3 beta
h1-h4: Hydrophobic residues 1–4
HER2: Human epidermal growth factor receptor 2
hPSCs: Human pluripotent stem cells
IFNa: Interferon alpha
ITD: Internal tandem duplications
MCL-1: Myeloid cell leukemia-1
MEK: Mitogen-activated protein kinase kinase
MM: Multiple myeloma
MOMP: Mitochondrial outer membrane permeabilization
mRNA: T-cell acute lymphoblastic leukemia
mTOR: Mechanistic target of rapamycin kinase
Mule: MCL-1 ubiquitin ligase E3
Myc: MYC proto-oncogene
NHL: Non-Hodgkin lymphoma
NOXA (PMAIP1): Phorbol-12-myristate-13-acetate-induced protein 1
NRAS: Neuroblastoma RAS viral (V-Ras) oncogene homolog
NSCLC: Non-small-cell lung cancer
OPA1: Optic atrophy 1
OS: Overall survival
OXPHOS: Oxidative phosphorylation
P1-P4: Four hydrophobic binding pockets 1 -4
PEST: Proline, glutamic acid, serine and threonine residues
PFS: Progression-free survival
PI3: Peptidase inhibitor 3
Pin1: Peptidylprolyl cis/trans isomerase, NIMA-interacting 1
PROTACs: Proteolysis-targeting chimeras
PTPN11: Protein tyrosine phosphatase non-receptor type 11
PUMA (BBC3): Bcl-2-binding component 3
RAS: Rat sarcoma
SCF: Skp1-cullin-F box
SCLC: Small-cell lung cancer
STAT3: Signal transducer and activator of transcription 3
SYK: Spleen-associated tyrosine kinase
T-ALL: T-cell acute lymphoblastic leukemia
TP53: Tumor protein P53
USP13,24, 9X: Ubiquitin-specific peptidase 13,24, 9 X-linked
VEGF: Vascular endothelial growth factor
β-TrCP: Beta-transducin repeat containing E3 ubiquitin protein ligase

## References

[1] Singh R, Letai A, Sarosiek K (2019). Regulation of apoptosis in health and disease: the balancing act of BCL-2 family proteins. Nat Rev Mol Cell Biol.

[2] Sarosiek KA (2017). Developmental regulation of mitochondrial apoptosis by c-myc governs age- and tissue-specific sensitivity to cancer therapeutics. Cancer Cell.

[3] Gutierrez-Martinez P (2018). Diminished apoptotic priming and ATM signalling confer a survival advantage onto aged haematopoietic stem cells in response to DNA damage. Nat Cell Biol.

[4] Sarosiek KA, Letai A (2016). Directly targeting the mitochondrial pathway of apoptosis for cancer therapy using BH3 mimetics - recent successes, current challenges and future promise. FEBS J.

[5] Deng J (2007). BH3 profiling identifies three distinct classes of apoptotic blocks to predict response to ABT-737 and conventional chemotherapeutic agents. Cancer Cell.

[6] Touzeau C (2016). BH3 profiling identifies heterogeneous dependency on Bcl-2 family members in multiple myeloma and predicts sensitivity to BH3 mimetics. Leukemia.

[7] Gong J-N (2016). Hierarchy for targeting prosurvival BCL2 family proteins in multiple myeloma: pivotal role of MCL1. Blood.

[8] Tsherniak A (2017). Defining a cancer dependency map. Cell.

[9] Kozopas KM, Yang T, Buchan HL, Zhou P, Craig RW (1993). MCL1, a gene expressed in programmed myeloid cell differentiation, has sequence similarity to BCL2. Proc Natl Acad Sci U S A.

[10] Petros AM, Olejniczak ET, Fesik SW (2004). Structural biology of the Bcl-2 family of proteins. Biochim Biophys Acta Mol Cell Res.

[11] Sattler M (1997). Structure of Bcl-xL-Bak peptide complex: recognition between regulators of apoptosis. Science.

[12] Denis C, Sopková-de Oliveira Santos J, Bureau R, Voisin-Chiret AS (2020). Hot-Spots of Mcl-1 Protein. J Med Chem.

[13] Senichkin VV, Streletskaia AY, Gorbunova AS, Zhivotovsky B, Kopeina GS (2020). Saga of Mcl-1: regulation from transcription to degradation. Cell Death Differ..

[14] Cl, D. *et al.* Solution structure of prosurvival Mcl-1 and characterization of its binding by proapoptotic BH3-only ligands. J Biol Chem 2005. 10.1074/jbc.M411434200 15550399

[15] Kale J, Osterlund EJ, Andrews DW (2018). BCL-2 family proteins: changing partners in the dance towards death. Cell Death Differ.

[16] Day CL (2008). Structure of the BH3 domains from the p53-inducible BH3-only proteins Noxa and Puma in complex with Mcl-1. J Mol Biol.

[17] Opferman JT (2003). Development and maintenance of B and T lymphocytes requires antiapoptotic MCL-1. Nature.

[18] Senichkin VV, Streletskaia AY, Zhivotovsky B, Kopeina GS (2019). Molecular comprehension of Mcl-1: from gene structure to cancer therapy. Trends Cell Biol..

[19] Rinkenberger JL, Horning S, Klocke B, Roth K, Korsmeyer SJ (2000). Mcl-1 deficiency results in peri-implantation embryonic lethality. Genes Dev.

[20] Wang X (2013). Deletion of MCL-1 causes lethal cardiac failure and mitochondrial dysfunction. Genes Dev.

[21] Perciavalle RM (2012). Anti-apoptotic MCL-1 localizes to the mitochondrial matrix and couples mitochondrial fusion to respiration. Nat Cell Biol.

[22] Rasmussen ML (2018). A Non-apoptotic Function of MCL-1 in Promoting Pluripotency and Modulating Mitochondrial Dynamics in Stem Cells. Stem Cell Reports.

[23] Rasmussen, M. L. *et al.* MCL-1 Inhibition by selective BH3 mimetics disrupts mitochondrial dynamics causing loss of viability and functionality of human cardiomyocytes. *iScience* **23**, (2020). 10.1016/j.isci.2020.101015 PMC7155208 32283523

[24] Chen G (2018). Targeting Mcl-1 enhances DNA replication stress sensitivity to cancer therapy. J Clin Invest.

[25] Germain M (2011). MCL-1 is a stress sensor that regulates autophagy in a developmentally regulated manner. EMBO J.

[26] Hanahan D, Weinberg RA (2011). Hallmarks of cancer: the next generation. Cell.

[27] AACR Project GENIE (2017). Powering precision medicine through an international consortium. Cancer Discov.

[28] Lv X (2015). Somatic mutations in myeloid cell leukemia-1 contribute to the pathogenesis of glioma by prolonging its half-life. Mol Med Rep.

[29] Beroukhim R (2010). The landscape of somatic copy-number alteration across human cancers. Nature.

[30] Campbell KJ (2018). MCL-1 is a prognostic indicator and drug target in breast cancer. Cell Death Dis.

[31] Zhou P (2001). MCL1 transgenic mice exhibit a high incidence of B-cell lymphoma manifested as a spectrum of histologic subtypes. Blood.

[32] Grabow S, Delbridge ARD, Aubrey BJ, Vandenberg CJ, Strasser A (2016). Loss of a single Mcl-1 allele inhibits MYC-driven lymphomagenesis by sensitizing pro-B cells to apoptosis. Cell Rep.

[33] Glaser SP (2012). Anti-apoptotic Mcl-1 is essential for the development and sustained growth of acute myeloid leukemia. Genes Dev.

[34] Grabow S, Delbridge ARD, Valente LJ, Strasser A (2014). MCL-1 but not BCL-XL is critical for the development and sustained expansion of thymic lymphoma in p53-deficient mice. Blood.

[35] Spinner S (2016). Re-activation of mitochondrial apoptosis inhibits T-cell lymphoma survival and treatment resistance. Leukemia.

[36] Schwickart M (2010). Deubiquitinase USP9X stabilizes MCL1 and promotes tumour cell survival. Nature.

[37] Wei G (2012). Chemical genomics identifies small-molecule MCL1 repressors and BCL-xL as a predictor of MCL1 dependency. Cancer Cell.

[38] Brunelle JK, Ryan J, Yecies D, Opferman JT, Letai A (2009). MCL-1-dependent leukemia cells are more sensitive to chemotherapy than BCL-2-dependent counterparts. J Cell Biol.

[39] Wertz IE (2011). Sensitivity to antitubulin chemotherapeutics is regulated by MCL1 and FBW7. Nature.

[40] Yeh C-H, Bellon M, Pancewicz-Wojtkiewicz J, Nicot C (2016). Oncogenic mutations in the FBXW7 gene of adult T-cell leukemia patients. Proc Natl Acad Sci U S A.

[41] Inuzuka H (2011). SCF(FBW7) regulates cellular apoptosis by targeting MCL1 for ubiquitylation and destruction. Nature.

[42] Min S-H (2012). Negative regulation of the stability and tumor suppressor function of Fbw7 by the Pin1 prolyl isomerase. Mol Cell.

[43] Peterson LF (2015). Targeting deubiquitinase activity with a novel small-molecule inhibitor as therapy for B-cell malignancies. Blood.

[44] Zhang S (2018). Deubiquitinase USP13 dictates MCL1 stability and sensitivity to BH3 mimetic inhibitors. Nat Commun.

[45] Lee K-M (2017). MYC and MCL1 cooperatively promote chemotherapy-resistant breast cancer stem cells via regulation of mitochondrial oxidative phosphorylation. Cell Metab.

[46] Wei G (2006). Gene expression-based chemical genomics identifies rapamycin as a modulator of MCL1 and glucocorticoid resistance. Cancer Cell.

[47] Elgendy M (2019). Combination of hypoglycemia and metformin impairs tumor metabolic plasticity and growth by modulating the PP2A-GSK3β-MCL-1 axis. Cancer Cell.

[48] Ni Chonghaile T (2011). Pretreatment mitochondrial priming correlates with clinical response to cytotoxic chemotherapy. Science.

[49] Nangia V (2018). Exploiting MCL1 dependency with combination MEK + MCL1 inhibitors leads to induction of apoptosis and tumor regression in KRAS-mutant non-small cell lung cancer. Cancer Discov.

[50] Comprehensive molecular profiling of lung adenocarcinoma. Nature **511**, 543–550 (2014). 10.1038/nature13385 PMC4231481 25079552

[51] Comprehensive genomic characterization of squamous cell lung cancers. Nature **489**, 519–525 (2012). 10.1038/nature11404 PMC3466113 22960745

[52] Song K-A (2018). Increased Synthesis of MCL-1 protein underlies initial survival of EGFR-mutant lung cancer to EGFR inhibitors and provides a novel drug target. Clin. cancer Res..

[53] Borner MM (1999). Expression of apoptosis regulatory proteins of the Bcl-2 family and p53 in primary resected non-small-cell lung cancer. Br J Cancer.

[54] Nakano T, Go T, Nakashima N, Liu D, Yokomise H (2020). Overexpression of antiapoptotic MCL-1 predicts worse overall survival of patients with non-small cell lung cancer. Anticancer Res.

[55] Inoue-Yamauchi A (2017). Targeting the differential addiction to anti-apoptotic BCL-2 family for cancer therapy. Nat Commun.

[56] Yasuda Y (2020). MCL1 inhibition is effective against a subset of small-cell lung cancer with high MCL1 and low BCL-X(L) expression. Cell Death Dis.

[57] Perillo B, Sasso A, Abbondanza C, Palumbo G (2000). 17beta-estradiol inhibits apoptosis in MCF-7 cells, inducing bcl-2 expression via two estrogen-responsive elements present in the coding sequence. Mol Cell Biol.

[58] Louault K (2019). Interactions between cancer-associated fibroblasts and tumor cells promote MCL-1 dependency in estrogen receptor-positive breast cancers. Oncogene.

[59] Ding Q (2007). Myeloid cell leukemia-1 inversely correlates with glycogen synthase kinase-3beta activity and associates with poor prognosis in human breast cancer. Cancer Res.

[60] Williams MM (2017). Key survival factor, Mcl-1, correlates with sensitivity to combined Bcl-2/Bcl-xL blockade. Mol Cancer Res.

[61] Balko JM (2014). Molecular profiling of the residual disease of triple-negative breast cancers after neoadjuvant chemotherapy identifies actionable therapeutic targets. Cancer Discov.

[62] Williams MM (2019). Therapeutic inhibition of Mcl-1 blocks cell survival in estrogen receptor-positive breast cancers. Oncotarget.

[63] Vallet S (2019). Rationally derived drug combinations with the novel Mcl-1 inhibitor EU-5346 in breast cancer. Breast Cancer Res Treat.

[64] Merino, D. *et al.* Synergistic action of the MCL-1 inhibitor S63845 with current therapies in preclinical models of triple-negative and HER2-amplified breast cancer. *Sci. Transl. Med.* **9**, (2017). 10.1126/scitranslmed.aam7049 28768804

[65] Lim SY, Menzies AM, Rizos H (2017). Mechanisms and strategies to overcome resistance to molecularly targeted therapy for melanoma. Cancer.

[66] Cook SJ, Stuart K, Gilley R, Sale MJ (2017). Control of cell death and mitochondrial fission by ERK1/2 MAP kinase signalling. FEBS J.

[67] Lee EF (2019). BCL-XL and MCL-1 are the key BCL-2 family proteins in melanoma cell survival. Cell Death Dis.

[68] Mukherjee N (2017). Use of a MCL-1 inhibitor alone to de-bulk melanoma and in combination to kill melanoma initiating cells. Oncotarget.

[69] McKee CS, Hill DS, Redfern CPF, Armstrong JL, Lovat PE (2013). Oncogenic BRAF signalling increases Mcl-1 expression in cutaneous metastatic melanoma. Exp Dermatol.

[70] Sale MJ (2019). Targeting melanoma’s MCL1 bias unleashes the apoptotic potential of BRAF and ERK1/2 pathway inhibitors. Nat Commun.

[71] Lee W-S (2015). Myeloid cell leukemia-1 is associated with tumor progression by inhibiting apoptosis and enhancing angiogenesis in colorectal cancer. Am J Cancer Res.

[72] Wilhelm SM (2008). Preclinical overview of sorafenib, a multikinase inhibitor that targets both Raf and VEGF and PDGF receptor tyrosine kinase signaling. Mol Cancer Ther.

[73] Yu C (2005). The role of Mcl-1 downregulation in the proapoptotic activity of the multikinase inhibitor BAY 43–9006. Oncogene.

[74] Tong J, Tan S, Zou F, Yu J, Zhang L (2017). FBW7 mutations mediate resistance of colorectal cancer to targeted therapies by blocking Mcl-1 degradation. Oncogene.

[75] Tong J (2017). Mcl-1 degradation is required for targeted therapeutics to eradicate colon cancer cells. Cancer Res.

[76] He K (2016). BRAFV600E-dependent Mcl-1 stabilization leads to everolimus resistance in colon cancer cells. Oncotarget.

[77] Kawakami H (2016). Mutant BRAF upregulates MCL-1 to confer apoptosis resistance that is reversed by MCL-1 antagonism and cobimetinib in colorectal cancer. Mol Cancer Ther.

[78] Lin L (2020). Trametinib potentiates TRAIL-induced apoptosis via FBW7-dependent Mcl-1 degradation in colorectal cancer cells. J Cell Mol Med.

[79] Song X (2020). Mcl-1 inhibition overcomes intrinsic and acquired regorafenib resistance in colorectal cancer. Theranostics.

[80] Slomp A, Peperzak V (2018). Role and regulation of pro-survival BCL-2 proteins in multiple myeloma. Front Oncol.

[81] Chonghaile TN (2014). Maturation stage of T-cell acute lymphoblastic leukemia determines BCL-2 versus BCL-XL dependence and sensitivity to ABT-199. Cancer Discov.

[82] Pei S (2020). Monocytic subclones confer resistance to venetoclax-based therapy in patients with acute myeloid leukemia. Cancer Discov.

[83] Peperzak V (2013). Mcl-1 is essential for the survival of plasma cells. Nat Immunol.

[84] Derenne S (2002). Antisense strategy shows that Mcl-1 rather than Bcl-2 or Bcl-x(L) is an essential survival protein of human myeloma cells. Blood.

[85] Zhang B, Gojo I, Fenton RG (2002). Myeloid cell factor-1 is a critical survival factor for multiple myeloma. Blood.

[86] Kumar S (2017). Efficacy of venetoclax as targeted therapy for relapsed/refractory t(11;14) multiple myeloma. Blood.

[87] Bajpai R (2020). Electron transport chain activity is a predictor and target for venetoclax sensitivity in multiple myeloma. Nat Commun.

[88] Touzeau C (2014). The Bcl-2 specific BH3 mimetic ABT-199: a promising targeted therapy for t(11;14) multiple myeloma. Leukemia.

[89] Wuillème-Toumi S (2005). Mcl-1 is overexpressed in multiple myeloma and associated with relapse and shorter survival. Leukemia.

[90] Gomez-Bougie P (2018). BH3-mimetic toolkit guides the respective use of BCL2 and MCL1 BH3-mimetics in myeloma treatment. Blood.

[91] Morales AA (2011). Distribution of Bim determines Mcl-1 dependence or codependence with Bcl-xL/Bcl-2 in Mcl-1-expressing myeloma cells. Blood.

[92] Seiller C (2020). Dual targeting of BCL2 and MCL1 rescues myeloma cells resistant to BCL2 and MCL1 inhibitors associated with the formation of BAX/BAK hetero-complexes. Cell Death Dis.

[93] Slomp A (2019). Multiple myeloma with 1q21 amplification is highly sensitive to MCL-1 targeting. Blood Adv.

[94] Jourdan M, De Vos J, Mechti N, Klein B (2000). Regulation of Bcl-2-family proteins in myeloma cells by three myeloma survival factors: interleukin-6, interferon-alpha and insulin-like growth factor 1. Cell Death Differ.

[95] Gupta VA (2017). Bone marrow microenvironment-derived signals induce Mcl-1 dependence in multiple myeloma. Blood.

[96] De Veirman, K. *et al.* Multiple myeloma induces Mcl-1 expression and survival of myeloid-derived suppressor cells. Oncotarget **6**, (2015). 10.18632/oncotarget.3300 PMC4496373 25871384

[97] Gomez-Bougie P, Oliver L, Le Gouill S, Bataille R, Amiot M (2005). Melphalan-induced apoptosis in multiple myeloma cells is associated with a cleavage of Mcl-1 and Bim and a decrease in the Mcl-1/Bim complex. Oncogene.

[98] Podar K (2008). A pivotal role for Mcl-1 in Bortezomib-induced apoptosis. Oncogene.

[99] Gomez-Bougie P (2007). Noxa up-regulation and Mcl-1 cleavage are associated to apoptosis induction by bortezomib in multiple myeloma. Cancer Res.

[100] Fan F (2014). Targeting Mcl-1 for multiple myeloma (MM) therapy: drug-induced generation of Mcl-1 fragment Mcl-1(128–350) triggers MM cell death via c-Jun upregulation. Cancer Lett.

[101] Tunquist BJ, Woessner RD, Walker DH (2010). Mcl-1 stability determines mitotic cell fate of human multiple myeloma tumor cells treated with the kinesin spindle protein inhibitor ARRY-520. Mol Cancer Ther.

[102] Davids MS (2017). Phase I first-in-human study of venetoclax in patients with relapsed or refractory non-hodgkin lymphoma. J Clin Oncol Off J Am Soc Clin Oncol.

[103] Hughes ME (2019). Treatment of patients with relapsed/refractory non-hodgkin lymphoma with venetoclax: a single-center evaluation of off-label use. Clin Lymphoma Myeloma Leuk.

[104] Prukova D (2019). Cotargeting of BCL2 with Venetoclax and MCL1 with S63845 Is Synthetically Lethal In Vivo in Relapsed Mantle Cell Lymphoma. Clin Cancer Res.

[105] Phillips DC (2015). Loss in MCL-1 function sensitizes non-Hodgkin’s lymphoma cell lines to the BCL-2-selective inhibitor venetoclax (ABT-199). Blood Cancer J.

[106] Smith VM (2020). Dual dependence on BCL2 and MCL1 in T-cell prolymphocytic leukemia. Blood Adv.

[107] Caenepeel S (2018). AMG 176, a selective MCL1 inhibitor, is effective in hematologic cancer models alone and in combination with established therapies. Cancer Discov.

[108] Smith VM (2020). Specific interactions of BCL-2 family proteins mediate sensitivity to BH3-mimetics in diffuse large B-cell lymphoma. Haematologica.

[109] Kotschy A (2016). The MCL1 inhibitor S63845 is tolerable and effective in diverse cancer models. Nature.

[110] Manzano M (2018). Gene essentiality landscape and druggable oncogenic dependencies in herpesviral primary effusion lymphoma. Nat Commun.

[111] Zhao S (2020). Efficacy of venetoclax in high risk relapsed mantle cell lymphoma (MCL) - outcomes and mutation profile from venetoclax resistant MCL patients. Am J Hematol.

[112] Kelly GL (2014). Targeting of MCL-1 kills MYC-driven mouse and human lymphomas even when they bear mutations in p53. Genes Dev.

[113] Kitada S (1998). Expression of apoptosis-regulating proteins in chronic lymphocytic leukemia: correlations with In vitro and In vivo chemoresponses. Blood.

[114] Bannerji R (2003). Apoptotic-regulatory and complement-protecting protein expression in chronic lymphocytic leukemia: relationship to in vivo rituximab resistance. J Clin Oncol Off J Am Soc Clin Oncol.

[115] Balakrishnan K (2014). Regulation of Mcl-1 expression in context to bone marrow stromal microenvironment in chronic lymphocytic leukemia. Neoplasia.

[116] Gobessi S (2009). Inhibition of constitutive and BCR-induced Syk activation downregulates Mcl-1 and induces apoptosis in chronic lymphocytic leukemia B cells. Leukemia.

[117] Bojarczuk K (2016). BCR signaling inhibitors differ in their ability to overcome Mcl-1-mediated resistance of CLL B cells to ABT-199. Blood.

[118] Roberts AW (2012). Substantial susceptibility of chronic lymphocytic leukemia to BCL2 inhibition: results of a phase I study of navitoclax in patients with relapsed or refractory disease. J Clin Oncol Off J Am Soc Clin Oncol.

[119] Klanova, M. & Klener, P. BCL-2 proteins in pathogenesis and therapy of B-cell non-hodgkin lymphomas. *Cancers (Basel).* **12**, (2020). 10.3390/cancers12040938 PMC7226356 32290241

[120] Chen R, Keating MJ, Gandhi V, Plunkett W (2005). Transcription inhibition by flavopiridol: mechanism of chronic lymphocytic leukemia cell death. Blood.

[121] Yi X (2020). AMG-176, an Mcl-1 antagonist, shows preclinical efficacy in chronic lymphocytic leukemia. Clin. cancer Res..

[122] Chua, C. C. *et al.* Chemotherapy and venetoclax in elderly acute myeloid leukemia trial (CAVEAT): a phase ib dose-escalation study of venetoclax combined with modified intensive chemotherapy. *J. Clin. Oncol. Off. J. Am. Soc. Clin. Oncol.* JCO2000572 (2020). doi:10.1200/JCO.20.00572 10.1200/JCO.20.00572 32687450

[123] Wei AH (2020). Venetoclax plus LDAC for newly diagnosed AML ineligible for intensive chemotherapy: a phase 3 randomized placebo-controlled trial. Blood.

[124] DiNardo CD (2020). Molecular patterns of response and treatment failure after frontline venetoclax combinations in older patients with AML. Blood.

[125] Yoshimoto G (2009). FLT3-ITD up-regulates MCL-1 to promote survival of stem cells in acute myeloid leukemia via FLT3-ITD-specific STAT5 activation. Blood.

[126] Breitenbuecher F (2009). A novel molecular mechanism of primary resistance to FLT3-kinase inhibitors in AML. Blood.

[127] Kaufmann SH (1998). Elevated expression of the apoptotic regulator Mcl-1 at the time of leukemic relapse. Blood.

[128] Li X-X (2019). Increased MCL-1 expression predicts poor prognosis and disease recurrence in acute myeloid leukemia. Onco Targets Ther.

[129] Kuusanmäki H (2020). Phenotype-based drug screening reveals association between venetoclax response and differentiation stage in acute myeloid leukemia. Haematologica.

[130] Ewald L, Dittmann J, Vogler M, Fulda S (2019). Side-by-side comparison of BH3-mimetics identifies MCL-1 as a key therapeutic target in AML. Cell Death Dis.

[131] Moujalled DM (2019). Combining BH3-mimetics to target both BCL-2 and MCL1 has potent activity in pre-clinical models of acute myeloid leukemia. Leukemia.

[132] Pan R (2015). Inhibition of Mcl-1 with the pan-Bcl-2 family inhibitor (-)BI97D6 overcomes ABT-737 resistance in acute myeloid leukemia. Blood.

[133] Lin KH (2016). Targeting MCL-1/BCL-XL forestalls the acquisition of resistance to ABT-199 in acute myeloid leukemia. Sci Rep.

[134] Fiskus W (2019). Superior efficacy of cotreatment with BET protein inhibitor and BCL2 or MCL1 inhibitor against AML blast progenitor cells. Blood Cancer J.

[135] Stevens BM (2018). PTPN11 mutations confer unique metabolic properties and increase resistance to venetoclax and azacitidine in acute myelogenous leukemia. Blood.

[136] Chen L (2015). Mutated Ptpn11 alters leukemic stem cell frequency and reduces the sensitivity of acute myeloid leukemia cells to Mcl1 inhibition. Leukemia.

[137] Czabotar PE (2007). Structural insights into the degradation of Mcl-1 induced by BH3 domains. Proc Natl Acad Sci U S A.

[138] Day CL (2005). Solution structure of prosurvival Mcl-1 and characterization of its binding by proapoptotic BH3-only ligands. J Biol Chem.

[139] Soderquist R, Eastman A (2016). BCL2 inhibitors as anticancer drugs: a plethora of misleading BH3 mimetics. Mol Cancer Ther.

[140] Gregory GP (2015). CDK9 inhibition by dinaciclib potently suppresses Mcl-1 to induce durable apoptotic responses in aggressive MYC-driven B-cell lymphoma in vivo. Leukemia.

[141] Wu X, Luo Q, Liu Z (2020). Ubiquitination and deubiquitination of MCL1 in cancer: deciphering chemoresistance mechanisms and providing potential therapeutic options. Cell Death Dis.

[142] Szlávik Z (2019). Structure-guided discovery of a selective MCL-1 inhibitor with cellular activity. J Med Chem.

[143] Brennan MS (2018). Humanized Mcl-1 mice enable accurate preclinical evaluation of MCL-1 inhibitors destined for clinical use. Blood.

[144] Halilovic E (2019). Abstract 4477: MIK665/S64315, a novel Mcl-1 inhibitor, in combination with Bcl-2 inhibitors exhibits strong synergistic antitumor activity in a range of hematologic malignancies. Cancer Res..

[145] Tron AE (2018). Discovery of Mcl-1-specific inhibitor AZD5991 and preclinical activity in multiple myeloma and acute myeloid leukemia. Nat Commun.

[146] Caenepeel S (2020). Abstract 6218: Discovery and preclinical evaluation of AMG 397, a potent, selective and orally bioavailable MCL1 inhibitor. Cancer Res..

[147] Ramsey HE (2018). A Novel MCL1 inhibitor combined with venetoclax rescues venetoclax-resistant acute myelogenous leukemia. Cancer Discov.

[148] Lee T (2019). Discovery of potent myeloid cell leukemia-1 (Mcl-1) inhibitors that demonstrate in vivo activity in mouse xenograft models of human cancer. J Med Chem.

[149] Lu X, Liu Y-C, Orvig C, Liang H, Chen Z-F (2019). Discovery of β-carboline copper(II) complexes as Mcl-1 inhibitor and in vitro and in vivo activity in cancer models. Eur J Med Chem.

[150] He Y (2020). Proteolysis targeting chimeras (PROTACs) are emerging therapeutics for hematologic malignancies. J Hematol Oncol.

[151] Papatzimas JW (2019). From inhibition to degradation: targeting the antiapoptotic protein myeloid cell leukemia 1 (MCL1). J Med Chem.

[152] Wang Z (2019). Proteolysis targeting chimeras for the selective degradation of Mcl-1/Bcl-2 derived from nonselective target binding ligands. J Med Chem.

[153] Spencer, A. *et al.* A phase 1, first-in-human study of AMG 176, a selective MCL-1 inhibitor, in patients with relapsed or refractory multiple myeloma. Clin. Lymphoma, Myeloma Leuk. **19**, e53–e54 (2019).

[154] Certo M (2006). Mitochondria primed by death signals determine cellular addiction to antiapoptotic BCL-2 family members. Cancer Cell.

[155] Foight GW, Ryan JA, Gullá SV, Letai A, Keating AE (2014). Designed BH3 peptides with high affinity and specificity for targeting Mcl-1 in cells. ACS Chem Biol.

