# Correction to: MCL-1 inhibitors, fast-lane development of a new class of anti-cancer agents

**DOI:** 10.1186/s13045-021-01043-z

**Published:** 2021-02-17

**Authors:** Arnold Bolomsky, Meike Vogler, Murat Cem Köse, Caroline A. Heckman, Grégory Ehx, Heinz Ludwig, Jo Caers

**Affiliations:** 1 Wilhelminen Cancer Research Institute, Wilhelminenspital, Vienna, Austria; 2 Institute for Experimental Cancer Research in Pediatrics, Goethe-University, Frankfurt, Germany; 3 Department of Clinical Hematology, GIGA-I3, University of Liège, CHU De Liège, 35, Dom Univ Sart Tilman B, 4000 Liège, Belgium; 4 Institute for Molecular Medicine Finland-FIMM, HiLIFE-Helsinki Institute of Life Science, iCAN Digital Cancer Medicine Flagship, University of Helsinki, Helsinki, Finland

## Correction to: J Hematol Oncol (2020) 13:173 10.1186/s13045-020-01007-9

The original article contains an incorrect affiliation for co-author, Meike Vogler. The correct affiliation can be viewed in this Correction article.
